# Identifying New Therapeutic Targets via Modulation of Protein Corona Formation by Engineered Nanoparticles

**DOI:** 10.1371/journal.pone.0033650

**Published:** 2012-03-19

**Authors:** Rochelle R. Arvizo, Karuna Giri, Daniel Moyano, Oscar R. Miranda, Benjamin Madden, Daniel J. McCormick, Resham Bhattacharya, Vincent M. Rotello, Jean-Pierre Kocher, Priyabrata Mukherjee

**Affiliations:** 1 Department of Biochemistry and Molecular Biology, Mayo Clinic College of Medicine, Rochester, Minnesota, United States of America; 2 Department of Chemistry, University of Massachusetts, Amherst, Massachusetts, United States of America; 3 Proteomics Research Center, Mayo Clinic College of Medicine, Rochester, Minnesota, United States of America; 4 Biomedical Statistics and Informatics, Mayo Clinic College of Medicine, Rochester, Minnesota, United States of America; 5 Department of Physiology and Biomedical Engineering, Mayo Clinic College of Medicine, Rochester, Minnesota, United States of America; 6 Mayo Clinic Cancer Center, Mayo Clinic College of Medicine, Rochester, Minnesota, United States of America; Chittaranjan National Cancer Institute, India

## Abstract

**Background:**

We introduce a promising methodology to identify new therapeutic targets in cancer. Proteins bind to nanoparticles to form a protein corona. We modulate this corona by using surface-engineered nanoparticles, and identify protein composition to provide insight into disease development.

**Methods/Principal Findings:**

Using a family of structurally homologous nanoparticles we have investigated the changes in the protein corona around surface-functionalized gold nanoparticles (AuNPs) from normal and malignant ovarian cell lysates. Proteomics analysis using mass spectrometry identified hepatoma-derived growth factor (HDGF) that is found exclusively on positively charged AuNPs (^+^AuNPs) after incubation with the lysates. We confirmed expression of HDGF in various ovarian cancer cells and validated binding selectivity to ^+^AuNPs by Western blot analysis. Silencing of HDGF by siRNA resulted s inhibition in proliferation of ovarian cancer cells.

**Conclusion:**

We investigated the modulation of protein corona around surface-functionalized gold nanoparticles as a promising approach to identify new therapeutic targets. The potential of our method for identifying therapeutic targets was demonstrated through silencing of HDGF by siRNA, which inhibited proliferation of ovarian cancer cells. This integrated proteomics, bioinformatics, and nanotechnology strategy demonstrates that protein corona identification can be used to discover novel therapeutic targets in cancer.

## Introduction

Gold nanoparticles (AuNPs) have been widely studied for their potential in disease therapeutics as targeting agents, drug delivery vehicles, and as therapeutic agents themselves [1], [2]. When nanoparticles are exposed to biological fluids via systemic administration or *ex vivo* incubation, they interact with proteins to form a biological coating on the AuNP to form a protein coating or corona. This corona dictates the biological response, as well as many physical properties, of the AuNP [3]. Identifying the contents of the protein corona can provide unique insight into the biological function of the AuNPs, including their biodistribution, clearance, and potential toxicity [4], [5],[6]–[9]. In addition to affecting nanoparticle behavior, the process of corona formation provides a tool for probing the local proteome [10]. As such, protein-covered nanoparticles may also provide insight into disease states and thereby help to identify new therapeutic targets.

Formation of the protein corona will depend on the nature of the interaction between the nanoparticles/conjugates and the biological fluids chosen [11], as dictated by the surface properties of the nanoparticles [12]. The nature of the interaction will depend mainly on i) the charge of the nanoconjugate, i.e., positive, negative, zwitterionic, or neutral and ii) the hydrophobicity/hydrophilicity of the conjugate. In this manuscript, we examine the nature of the protein corona captured by surface-functionalized AuNPs when exposed to clinically relevant biological fluids, and reveal that tuning the nanoparticle surface charge can preferentially enrich and therefore enable detection of otherwise undetected low-abundance proteins as possible therapeutic targets. We used a combination of UV-Visible spectroscopy (UV-Vis), dynamic light scattering (DLS), zeta potential measurement, and Bradford protein assay to characterize the protein corona. Proteomics/bioinformatics analysis was used to identify low-abundance proteins only found when enriched on the nanoparticle surface. We incubated AuNPs with normal and malignant ovarian cell lysates using positively and negatively charged AuNPs (^+^AuNPs and ^−^AuNPs, respectively). In this study, we established HDGF as a possible therapeutic target for ovarian cancer by use of proteomic analysis and expression levels in cancer cells. This approach was validated through inhibition of cell proliferation upon silencing this factor by siRNA. Taken together, we show that this technique can be used to identify and validate new therapeutic targets for ovarian cancer, providing a generalized strategy for potential use in other diseases.

## Results

### Characterization of protein corona around surface-modified AuNPs from the lysates of normal OSE (Ovarian Surface Epithelial) and malignant serous ovarian cells (OV167)

**Figure 1:**
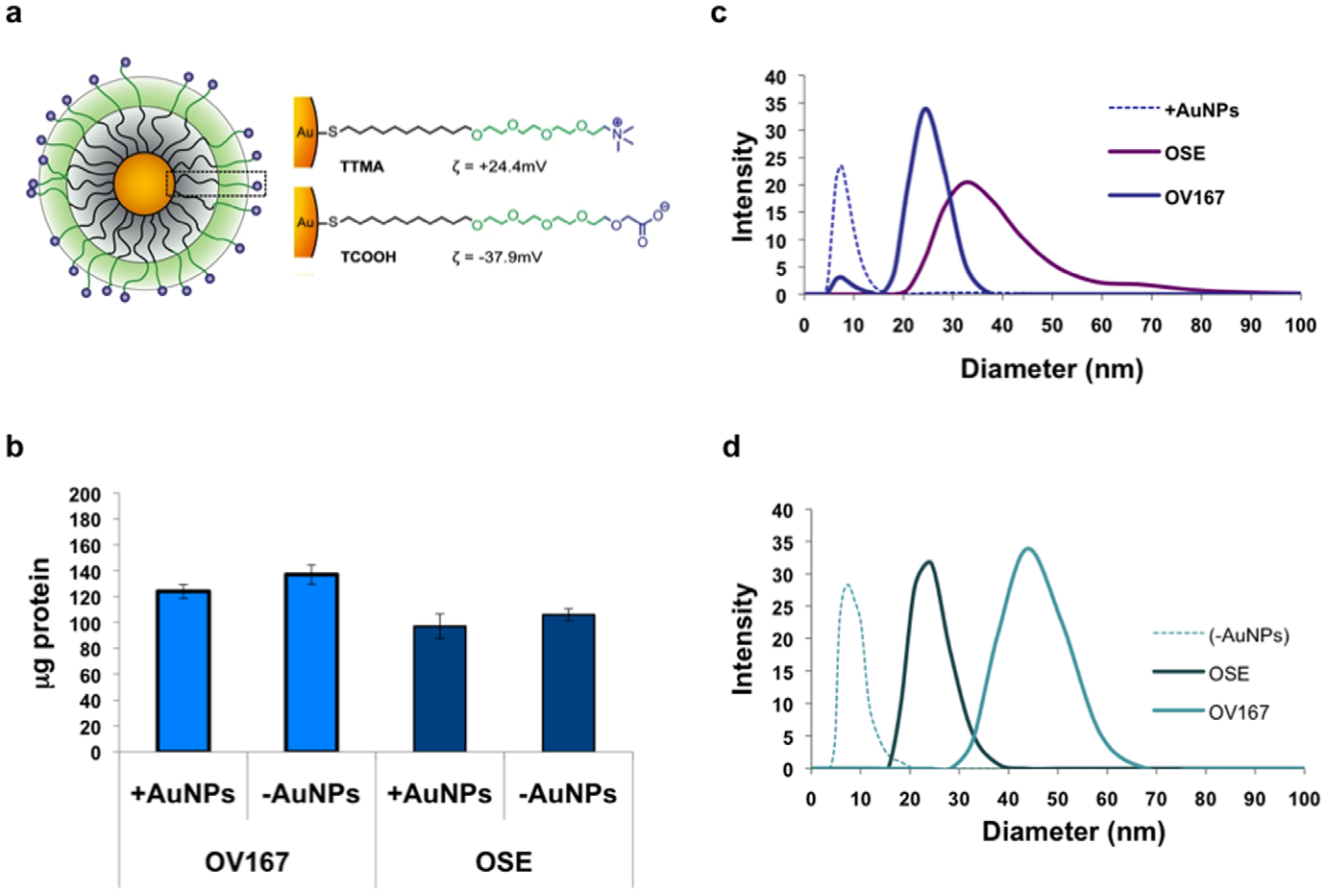
Characterization of the AuNP and protein corona made from cell lysates of OSE and OV167. a) A cartoon showing functionalization of 5 nm gold nanoparticle to create positively charged (^+^AuNP) or negatively charged (^−^AuNP) gold nanoparticles. b) Amount of protein bound on the nanoparticle as determined by Bradford assay. The binding of protein is evident from the increase in surface size via DLS on c) ^+^AuNP and d) ^−^AuNP.

Lysates were collected from ovarian cell lines, OV167 (malignant) [13] and OSE (non-cancerous) [14] were incubated with ^+^AuNPs and ^−^AuNPs to form protein coronas. By subtracting the amount of protein in the supernatants from the amount of protein in the non-gold control (equal to the molar amount of lysate in the buffer solution) the amount of protein adsorbed onto the AuNP surface was determined (Figure 1A). In these studies, 137 µg protein from the OV 167 cell lysate per 50 µL nanoparticle adsorbed onto the ^−^AuNP surface as opposed to 106 µg from the OSE lysate nanoparticle (Figure 1B). Similar amounts of protein also adsorbed onto ^+^AuNP (124 µg and 97 µg from OV 167 and OSE lysates, respectively). The size of the particles was examined by DLS before and after incubation with cell lysates (Figure 1C and 1D). The hydrodynamic diameter (d_H_) of ^+^AuNP, increased from 10 nm (Z-average) for the two nanoparticles prior to incubation(^+^AuNP and ^−^AuNP) with a Gaussian distribution (Table 1) to 32 and 24 nm after incubation with OV167 and OSE lysates, respectively (Figure 1C). For ^−^AuNP, d_H_ increased from 10 nm to 28 and 43 nm after incubation with OV167 and OSE lysates, respectively (Figure 1D). Upon formation of the protein corona, the zeta potential of ^−^AuNPs decreased from −44 mV to −25 mV (OV167) and −30 mV (OSE), respectively. Similarly, incubation with OV167 and OSE lysates decreased the zeta potential of ^+^AuNP from +25 mV to +5 mV and −3 mV, respectively (see Table 2). These data indicate that ^+^AuNPs and ^−^AuNPs both form different protein coronas from malignant and normal cell lysates and both lead to increased hydrodynamic diameter and a change in overall charge.

**Table 1:** Dynamic Light Scattering (DLS) to determine hydrodynamic diameter (d_H_) of AuNPs before and after incubation with OV167 and OSE lysates.

	^+^AuNP (d_H_)	^−^AuNP (d_H_)
Particle only	10 nm	10 nm
OV167	32	23 nm
OSE	24	43 nm

**Table 2:** Zeta Potential in mV of ^+^AuNP and ^−^AuNP before and after incubation with the lysates of OV167 and OSE, respectively.

	^+^AuNP	^−^AuNP
Particle only	+25±5	−45±10
OV167	+5±3	−25±8
OSE	−3±2	−30±11

### Validating reproducibility of corona formation around surface-modified AuNPs

**Figure 2:**
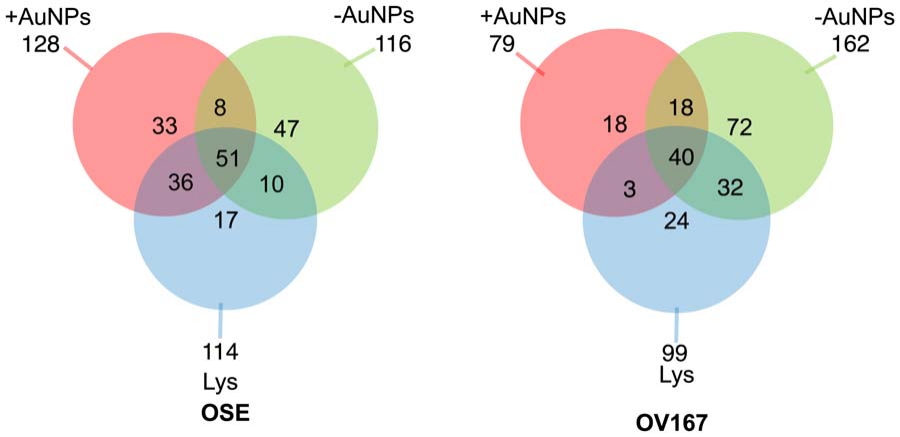
Selectivity of the proteins bound to positively charged (^+^AuNP) vs negatively charged (^−^AuNP) particles. Venn diagrams show proteins identified in the protein corona around ^+^AuNP and ^−^AuNP from a) normal OSE cell lysates and b) malignant OV167 cell lysates. The figure clearly depicts the preferential enrichment of low abundance proteins by engineered nanoparticles that were not detectable in the lysates by proteomics analysis. These proteins, which were otherwise undetected, could potentially be new therapeutic targets.

The protein coronas formed with ^+^AuNP and ^−^AuNP nanoparticles and the OV167 and OSE lysates were analyzed and identified by means of mass spectrometry. The relationship of the proteins identified from the lysate-formed coronas of ^+^AuNP and ^−^AuNP is shown in Figure 2 (see also Figure S1, Figure S2, Figure S3 and Tables S1, S2, S3, S4, S5, S6, S7, S8, S9, S10). Bioinformatics analysis of the proteins identified by proteomics studies from OSE lysates revealed a number of proteins that were uniquely found in the coronas of ^−^AuNP (47) and ^+^AuNP (33), (Figure 2A and Table S1). Similar analysis of the corona proteins from OV167 lysates showed that unique proteins were in the formed coronas of ^+^AuNPs (18) and ^−^AuNPs (72), respectively (Figure 2B and Table S2). It is important to note that the unique proteins are exclusive to ^+^AuNP and ^−^AuNP and not found at detectable levels in the corresponding cell lysates. These data clearly demonstrate the concentrating effect of protein corona formation upon low-abundance proteins. The identification of all the proteins is provided in the Supplementary material (see Tables S1, S2, S3, S4, S5, S6, S7, S8, S9, S10).

### Identification of new molecular targets from the protein corona formed on surface-modified AuNPs

**Figure 3:**
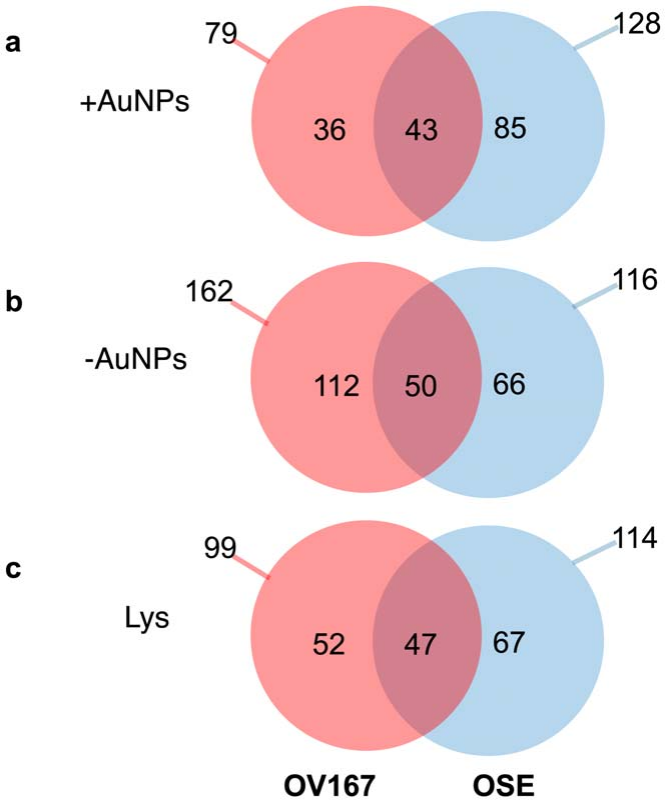
Venn diagram demonstrating the difference between the proteins that make up the corona for a) ^+^AuNP, b) ^−^AuNP from the cell lysates and c) the different between proteins in the OV167 and OSE cell lysates. These differences in the composition of the lysates could be exploited to identify new therapeutic targets in ovarian cancer.

**Figure 4:**
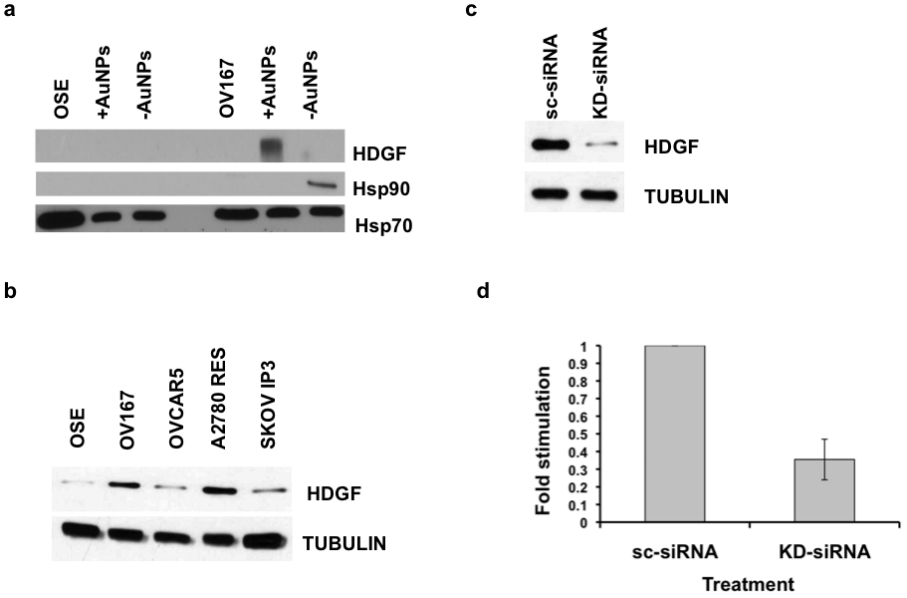
Enrichment and functional consequence of HDGF. a) Western blot confirming the presence of HDGF on ^+^AuNP- corona whereas Hsp90 on ^−^AuNP- corona. Also shown is Hsp70 (known to be present in all NP coronas via MS). b) Expression of HDGF in ovarian cell lines. c) Knockdown of HDGF in A2780 cell line using HDGF-siRNAs (KD-siRNA) and compared with the scrambled control (scRNA); d) Effect of silencing HDGF on proliferation of ovarian cancer cells analyzed by [3H]-thymidine incorporation assay.

We next compared the selectivity of each nanoparticle in discerning different proteins from OSE and OV167 cell lysates, as shown in Figure 3. A closer examination of our results showed that there was neither a distinct functional class nor a particular range of pIs in which the proteins bound to the nanoparticles (data not shown), which is consistent with prior findings [15]. Based on the selectivity, we investigated whether modulation of protein-corona formation by surface-functionalized nanoparticles could be applied to discover new therapeutic targets for cancer. Proteomics/bioinformatics analysis revealed hepatoma-derived growth factor (HDGF) to be a component of the ^+^AuNP protein corona from the malignant OV167 cells; thus, this was selected as a possible target. HDGF has recently been implicated in the pathogenesis of several other cancers but its role in ovarian cancer remains unknown so far. To further validate the specificity of ^+^AuNP and ^−^AuNP towards preferential enrichment of low abundance proteins, we selected Hsp70, Hsp90 and HDGF. As mentioned earlier HDGF and Hsp90 were only present in ^+^AuNP and ^−^AuNP corona of OV167 proteome, respectively. Hsp70 was used as a control due to its commonality between OSE and OV 167 lysates as well as to the protein corona formed on both ^+^AuNP and ^−^AuNP. Western blot analysis shows that HDGF is indeed preferentially enriched on the ^+^AuNPs, whereas it is not detected either in the lysates or on ^−^AuNPs, confirming the results from tandem MS. Similarly, Hsp90 is preferentially detected on ^−^AuNPs, whereas Hsp70 is detected in all the fractions (Figure 4A).

We then investigated the functional consequences of the presence of HDGF in ovarian cancer. We first determined the expression levels of HDGF in various ovarian cancer cell lines. As seen in the Western blot, HDGF is expressed across various ovarian cancer cell lines, with the highest expression in A2780 cell line [16] and the lowest in normal OSE (Figure 4B). To demonstrate functional effects we knocked down HDGF in A2780 cells, by using siRNA (KD-siRNA) and looked at the proliferation of the ovarian cancer cells. We chose A2780 cells because they expressed the highest level of HDGF of the cells tested. The silencing of HDGF was confirmed by Western blot analysis, and there was a convincing level of significant knockdown as compared to the scrambled control (Figure 4C). In addition, silencing of HDGF results in significant inhibition of proliferation of ovarian cancer cells (∼70%) as compared to the scrambled siRNA (sc-siRNA) control (Figure 4D). These results verify that HDGF is a potential therapeutic target in ovarian cancer.

## Discussion

Ovarian cancer is the most common malignancy of the female genital tract and one of the most lethal [17],[18], essentially because there are currently no early screening or diagnostic tests for this disease. As a result, the cancer often remains clinically undetected until the later stages of the disease [19]. Even though patients respond initially to chemotherapy after surgical debulking, most of them develop terminal drug-resistant relapse [20]. Hence, new therapeutic strategies, and thus new targets, are urgently needed to combat ovarian cancer. Identifying constituents of the protein corona that is formed when metal nanoparticles are incubated with cell lysates by using a combination of proteomics, bioinformatics, and nanotechnology could provide useful information regarding the development of the disease and allow us to discover new therapeutic targets in ovarian cancer, as well as potentially for other cancers in the future.

Quantitative proteomics has allowed researchers to study protein populations found in tissues or biofluids [21] [22] [23]. One technique used to study these protein sets is mass spectrometry, a detection tool with the ability to screen for myriad of proteins [24]. In general, mass spectrometry-based proteomics is used to interpret the information encoded from genomes [25]. Protein analysis by mass spectrometry has been fairly reliable when applied to small sets of protein samples [26] [27]. However, low-abundance proteins may escape detection using these general methodologies due to the Vroman effect, in which highly mobile proteins that adsorb onto surfaces are replaced by less mobile proteins [28],[29], [30]. In the present study, we were able to differentially detect proteins not observed in the cell lysates using conventional methods. In our strategy, these low abundance proteins were enriched by selective interaction with the modified surface of gold nanoparticles. The different proteins that adsorb onto the surface of the nanoparticle are reflected in protein corona light-scattering behavior, charge, and proteomics. These results clearly suggest that tuning the surface charge of engineered nanoparticles can modulate the formation of protein corona around them. Such modulation is important to detect low-abundance proteins in order to identify new therapeutic targets.

Recently there has been substantial interest in understanding the interactions between proteins and nanoparticles in various bio-fluids and their biophysical properties with respect to drug delivery and therapeutics. To this end, research groups have intently focused on determining the composition of the nanoparticle corona with human plasma [3], [7], [12], [15], [31], [32], demonstrating that nanoparticle surface size and properties play a critical role in protein adsorption [33]–[35]. Subsequent work also suggests that the protein corona is important for how the cell perceives and transports the nanoparticles [36]. In parallel to gaining knowledge in the selective affinity of nanoparticles for proteins, research has also focused on coating the surface of nanoparticles to detect specific antigens and protein sequences [34], [37]–[39]. The study presented here uses nanoparticles as useful tools for enrichment and detection of low abundance proteins as a unique approach to discover new therapeutic targets. Using bioinformatics, we were able to further distinguish proteins that are uniquely adsorbed to either ^+^AuNPs or ^−^AuNPs; these were validated via Western blot analysis. One of the proteins we chose to examine more closely is HDGF, which was identified as a *possible* ovarian-cancer-specific mitogen because it was found only with the lysates of the malignant cells, and only on the ^+^AuNPs. To date, it has not been established if HDGF has a role in ovarian cancer. However, it has been implicated in other cancers, such as melanoma, pancreatic, and lung [40]–[42]. From our studies, we showed that in comparison to OSE, HDGF is greatly over expressed in OV 167 and A2780 cell lines. Upon knockdown of HDGF expression, we observed that the proliferation of ovarian cancer cells was vastly reduced. These results correlate strongly with previous studies which showed that HDGF is co-expressed with proliferating cell nuclear antigen (PCNA) in smooth muscle cells [43]. These results demonstrate, for the first time, a possible role of HDGF in ovarian cancer pathogenesis and present it as a potential therapeutic target. They also introduce a new methodology of exploiting nanoparticle corona content to identify potential therapeutics.

In summary, this paper employs a unique combination of tools: proteomics, bioinformatics, and nanotechnology, to open up a new avenue for identifying and validating new therapeutic targets in cancer. Using surface-modified gold nanoparticles, we successfully enriched low-abundance proteins from lysates of normal and malignant ovarian cancer cells and identified them using a combination of proteomics and bioinformatics analysis. Although we focused on HDGF in this study, extension of similar methods to other protein targets to target other diseases would be valid. Future studies will focus on the evolution, modulation, and identification of protein coronas over time and also on testing this unique system with human patient samples (blood, serum, plasma). This study opens up a new area of research to unravel new signaling networks, not only for cancer but also for other diseases, by detecting low-abundance undetectable proteins using enrichment induced by surface-engineered nanoparticles.

## Materials and Methods

Gold salt was purchased from Strem Chemicals (Newburyport, MA). PBS,RPMI and DMEM was purchased from Mediatech, (Manassas, VA). HDGF antibody was purchased from Santa Cruz Biotechnologies (Santa Cruz, CA) and tubulin was purchased from AbCam (Cambridge, MA). Secondary antibodies were purchased from Santa Cruz. Antibodies to HSP70 and HSP90 were a gift from Dr. Scott Kaufmann.

### Particle synthesis

Gold nanoparticles were synthesized according to methods reported by You et al [44]. In brief, AuNPs were synthesized via the one-pot protocol developed by Schiffrin et al [45]. Further functionalization was performed using the Murray place-exchange method [46], with our thiolated ligands bearing an amine or carboxylate end group [47].

### Cell Lysates

Cells were grown using standard conditions. A2780 cells were grown using RPMI media with 10% FBS and 1% antibiotic/antimycotic. OSE cells grown in were medium 199 with Earle's salts (Sigma, St. Louis, USA) and MCDB 202 (Sigma), 1∶1 ratio, with 15% Fetal Bovine Serum (FBS). OV167, OVCAR5, and SKOV IP3 were grown in DMEM (10% FBS and 1% antibiotic/antimycotic). For each cell line, 2×150 mm dishes were grown to confluence (∼90%). Prior to lysing, the plates were rinsed five times with 1× phosphate-buffered saline (PBS) buffer to help reduce the amount of fetal bovine serum. Cells were then lysed using Ripa buffer (1 mL) with protein inhibitor cocktail (10 mL added) on ice for 30 minutes. The lysates were scraped off the plate and collected into a mini-centrifuge tube. The tubes were centrifuged at 14,000 rpm for 12 minutes at 4°C (twice) and the supernatants were collected. Cell lysates were kept at −20°C until needed.

### Corona Formation

Gold nanoparticles (1.2 µM) were incubated for 1 hour with 0.2 mg/mL of cell lysates at room temperature. The lysate/nanoparticle mixture was subjected to centrifugation (100,000× *g*/45 min/10°C) and the supernatant collected. The nanoparticle-corona pellet was washed with 1 mL of water and resuspended and re-centrifuged (100,000× *g*/45 min/10°C) and the resulting supernatant and pellets collected. The supernatants were measured for protein content via Bradford assay. The pellets were further analyzed for protein identification using MS-MS.

### DLS and Zeta Potential Analysis

A Malvern Zetasizer Nano ZS instrument was used to measure zeta potential at 25°C for all nanoparticles. Samples were prepared as mentioned above. Samples were loaded into a pre-rinsed folded capillary cell for the zeta potential and DLS measurements. The principle employed was ELS (Smoluchowski methodology for aqueous media) in a Zetasizer Nano ZS instrument. A maximum of 100 sub runs was performed until a constant value was obtained.

### Western Blot Analysis

Nanoparticle-protein corona was centrifuged as described above. The supernatant was separated from the pellet. The pellet was resuspended in 30 µl of double distilled H20 along with 25% Laemmeli's solution and 2.5% 2-βmercaptoethanol. The mixture was boiled at 95°C for 15 minutes and centrifuged at 4°C at 16500 rpm for 15 minutes. The supernatant is separated from the resulting pellet and 30 µl loaded on to gels. As for the control, 30 µl of the cell lysate was aliquoted from 200 µg/mL of lysate solution and boiled with 25% Laemmeli's solution at 95°C for 5 minutes. The proteins were run on a 10% gel and transferred to a PVDF membrane at 100V for 40 minutes. The membrane was blocked with 4% milk and incubated with primary antibodies overnight.

### siRNA Knockdown

A2780 cells were plated in 60-mm dishes with 3 mL cell culture media. Cells were transfected with 20 µL of 20 µM siRNA purchased from Qiagen (Valencia, CA) along with 20 µL of HiPerfect (Valencia, CA) and 500 µL of OPTI-MEM (Invitrogen, Carlsbad, CA). Scrambled siRNA from Qiagen was used as a control. After 48 hours, cells were counted and plated on 24-well plates (3×10^4^ cells/mL) containing 1 mL media. Cell lysate was also collected for Western blot analysis. 20 µg of whole-cell lysates were used to detect the knockdown efficiency of the siRNA.

### ^3^H-Thymidine incorporation assay for cellular proliferation

Cells transfected with siRNA were seeded (2×10^4^) in 24-well plates. After overnight incubation under standard conditions, 1 µCi/ml [3H]-thymidine was added to fresh media, and 4 h later, cells were washed with ice-cold PBS, fixed with 100% cold methanol followed by washing and lysing with 250 µl 0.1N NaOH. [3H]-thymidine incorporation in the cells was measured in a scintillation counter.

### Mass Spectrometry; Protein identification via trypsin digest → nanoLC-MS/MS with hybrid orbitrap/linear ion trap mass spectrometry

The nanoparticle solutions were prepared for mass spectrometry analysis using the following procedures. The solutions were dried on a SpeedVac spinning concentrator (Savant Instruments, Holbrook NY), then solubilized in 50 mM Tris/0.25% Protease Max (Promega Corporation, Madison, WI). The proteins were reduced and alkylated with tris(2-carboxyethyl)phosphine (TCEP) at 55°C for 40 minutes and iodoacetamide at room temperature for 40 min in the dark, then digested with trypsin (Promega Corporation, Madison WI) in 50 mM Tris pH 8.1/0.0002% Zwittergent 3–16, at 37°C overnight. The digest was terminated with 2% trifluoroacetic acid, and acetonitrile was added, then centrifuged to pellet the particles. The supernatant was removed and concentrated to less than 5 µL on a SpeedVac spinning concentrator (Savant Intruments, Holbrook NY). The digest mixture was brought up in 0.2% trifluoroacetic acid for protein identification by nano-flow liquid chromatography electrospray tandem mass spectrometry (nanoLC-ESI-MS/MS) using a ThermoFinnigan LTQ Orbitrap Hybrid Mass Spectrometer (Thermo Fisher Scientific, Bremen, Germany) coupled to an Eksigent nanoLC-2D HPLC system (Eksigent, Dublin, CA). The peptide digest mixture was loaded onto a 250 nL OPTI-PAK trap (Optimize Technologies, Oregon City, OR) custom-packed with Michrom Magic C8 solid phase (Michrom Bioresources, Auburn, CA). Chromatography was performed using 0.2% formic acid in both the A solvent (98% water/2% acetonitrile) and B solvent (80% acetonitrile/10% isopropanol/10% water), and a 5%B to 40%B gradient over 100 minutes at 325 nL/min through a hand-packed PicoFrit (New Objective, Woburn, MA) 75 µm×200 mm column (Michrom Magic C18, 3 µm). The LTQ Orbitrap mass spectrometer experiment was set to perform a Fourier-transformed full scan from 375–1600 m/z with resolution set at 60,000 (at 400 m/z), followed by linear ion trap MS/MS scans on the five most abundant ions. Dynamic exclusion was set to 1 and selected ions were placed on an exclusion list for 30 seconds. The lock-mass option was enabled for the FT full scans using the ambient air polydimethylcyclosiloxane (PCM) ion of m/z = 445.120024 or a common phthalate ion m/z = 391.284286 for real-time internal calibration [48].

### Mass Spectrometry; Protein and Peptide Identification

Tandem mass spectra were extracted by using BioWorks version 3.2. All MS/MS samples were analyzed using Mascot (Matrix Science, London, UK; version 2.2.04), Sequest (ThermoFinnigan, San Jose, CA; version 27, rev. 12) and X! Tandem (www.thegpm.org; version 2006.09.15.3). All the three applications identify peptides by comparing the experimental spectra to predicted spectra of peptide derived from the protein sequence available in the Swissprot database (699052 entries), assuming trypsin digestion. For the three applications the fragment ion mass tolerance was set to 0.80 Da. Parent ion tolerance was respectively set to 10.0 PPM for Mascot and X! Tandem and 0.0084 Da for Sequest. Oxidation of methionine and iodoacetamide derivative of cysteine were specified as variable modifications in Mascot, Sequest and X! Tandem. The applications returned the list of identified peptides, associated proteins, and confidence score. Scaffold (version Scaffold_2_00_06, Proteome Software Inc., Portland, OR) was used to combine the output of Mascot, X!Tandem and Sequest to enhance protein identifications. Confidence scores were combined in a probability model as specified in the PeptideProphet algorithm [49]; this model was used to score each peptide. Peptides with a probability score lower than 95.0% were rejected from the analysis. Proteins were then scored by an algorithm included in PeptideProphet [50] that also used a statistical model. Proteins that were associated with the same set of peptides and therefore could not be further discriminated were jointly associated to these peptides.

### Bioinformatics

The Mascot output is further processed by a set of bioinformatics procedures. Output files are summarized at the protein level to include one protein per entry. Since peptides can be related to several member of a protein family, only proteins with the extension ‘_HUMAN’ were retained during the processing; those not related to human proteins were rejected. The sequence of each protein was extracted from the Uniprot database (http://www.uniprot.org/ version May 15,2011). Protein comparison was performed based on the sequence of each protein rather than its name.

## Supporting Information

Figure S1 **A combination of MS and bioinformatics tools was used to identify proteins bound to the AuNPs surface from the OSE lysate.** Venn diagram shows the reproducibility of the identification process with triplicate MS runs for proteins associated with the different AuNPs.
(TIF)

Figure S2 **A combination of MS and bioinformatics tools was used to identify proteins bound to the AuNPs surface from the OV167 lysate.** Venn diagram shows the reproducibility of the identification process with triplicate MS runs for proteins associated with the different AuNPs.
(TIF)

Figure S3 **Characterization of the AuNP and protein corona made from cell lysates of a) normal ovarian cell line (OSE) and b) ovarian cancer cell line (OV167).** The binding of protein is evident from the increase in UV absorbance.
(TIF)

Table S1 **Unique Proteins present in the corona of ^+^AuNP and ^−^AuNP from OSE lysate.**
(DOCX)

Table S2 **Unique proteins present in the corona of ^+^AuNP and ^−^AuNP from OV167 lysate.**
(DOCX)

Table S3 **All Proteins OSE Lysate.**
(DOCX)

Table S4 **All proteins present in the corona of ^+^AuNP from OSE lysates.**
(DOCX)

Table S5 **All proteins present in the corona of ^−^AuNP from OSE lysates.**
(DOCX)

Table S6 **All proteins present in OV167 lysate.**
(DOCX)

Table S7 **All proteins in the OV167 ^+^AuNP corona.**
(DOCX)

Table S8 **All proteins in the OV167 ^−^AuNP corona.**
(DOCX)

Table S9 **Comparison of proteins present in ^−^AuNP corona from OSE and OV167 lysates.**
(DOCX)

Table S10 **Comparison of proteins present in ^+^AuNP corona from OSE and OV167 lysates.**
(DOCX)
