# Supplementary figures and images for: Identifying New Therapeutic Targets via Modulation of Protein Corona Formation by Engineered Nanoparticles
Source: PLoS One. 2012 Mar 19;7(3):e33650. doi: 10.1371/journal.pone.0033650 (PMC3307759; doi:10.1371/journal.pone.0033650)

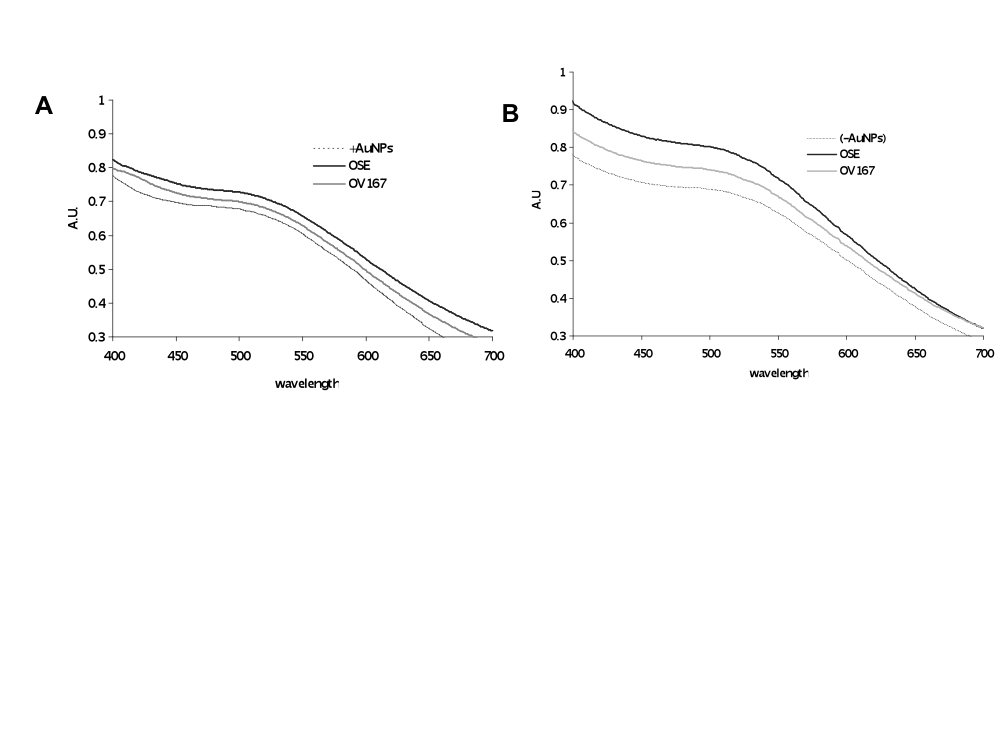

Supplement: Figure S1 — A combination of MS and bioinformatics tools was used to identify proteins bound to the AuNPs surface from the OSE lysate. Venn diagram shows the reproducibility of the identification process with triplicate MS runs for proteins associated with the different AuNPs. (TIF) [file pone.0033650.s001.tif]

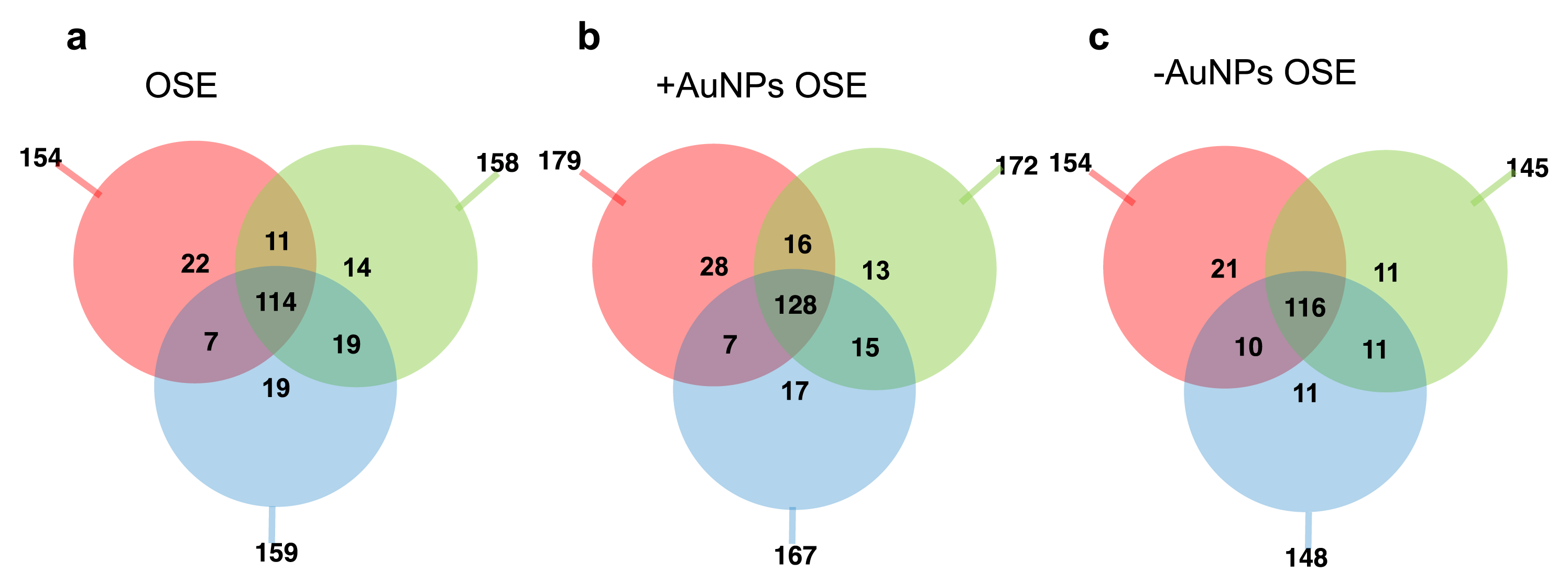

Supplement: Figure S2 — A combination of MS and bioinformatics tools was used to identify proteins bound to the AuNPs surface from the OV167 lysate. Venn diagram shows the reproducibility of the identification process with triplicate MS runs for proteins associated with the different AuNPs. (TIF) [file pone.0033650.s002.tif]

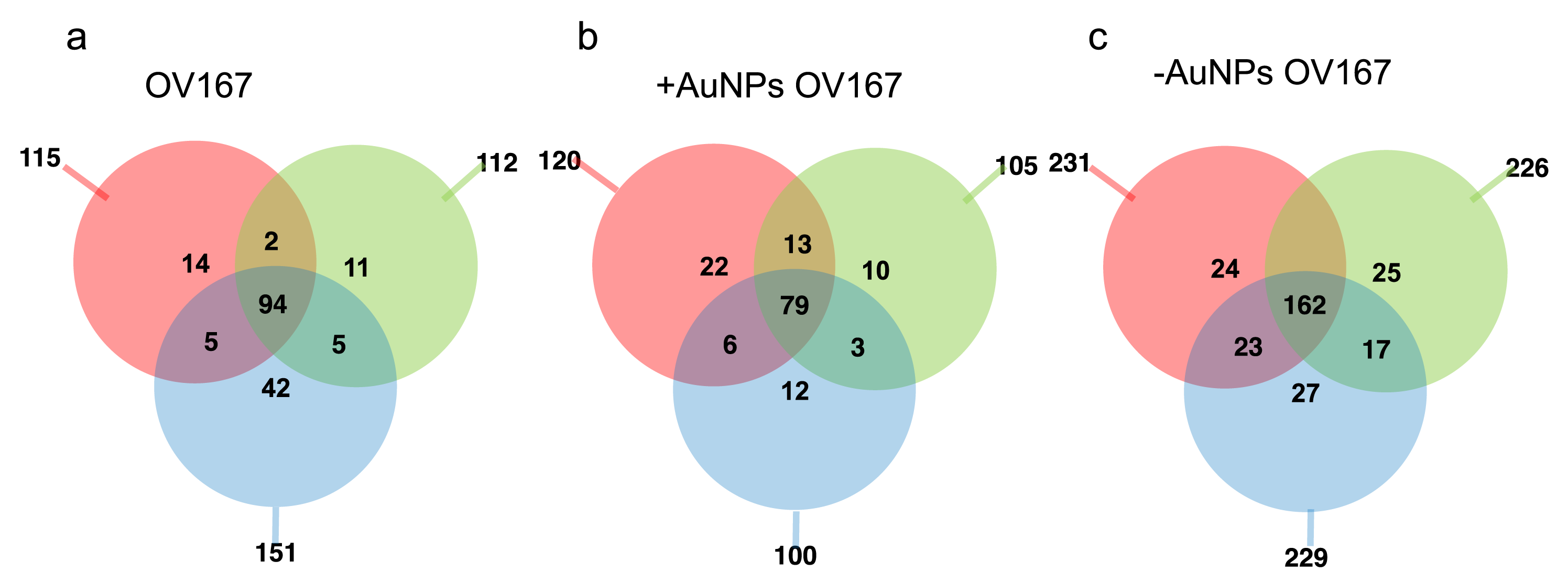

Supplement: Figure S3 — Characterization of the AuNP and protein corona made from cell lysates of a) normal ovarian cell line (OSE) and b) ovarian cancer cell line (OV167). The binding of protein is evident from the increase in UV absorbance. (TIF) [file pone.0033650.s003.tif]
