# Supplementary material for: Identifying New Therapeutic Targets via Modulation of Protein Corona Formation by Engineered Nanoparticles
Source: PLoS One. 2012 Mar 19;7(3):e33650. doi: 10.1371/journal.pone.0033650 (PMC3307759; doi:10.1371/journal.pone.0033650)
Supplement: Table S1 — Unique Proteins present in the corona of +AuNP and −AuNP from OSE lysate. (DOCX) [file pone.0033650.s004.docx]

**Table S1 Unique Proteins present in the corona of ^+^AuNP and ^–^AuNP from OSE lysate.**

| **Unique to ^+^AuNP** | | **Unique to ^-^AuNP** | |
| --- | --- | --- | --- |
| Gene Name | Full Name | Gene Name | Full Name |
| AT1A1_HUMAN | Sodium/potassium-transporting ATPase subunit alpha-1 | 1433G_HUMAN | Protein kinase C inhibitor protein 1 |
| C1QBP_HUMAN | GC1q-R protein | ATPO_HUMAN | ATP synthase subunit O, |
| CALU_HUMAN | Calumenin | CAV1_HUMAN | Caveolin-1 |
| CAPR1_HUMAN | Caprin-1 | CD44_HUMAN | CD44 antigen |
| CO1A1_HUMAN | Collagen alpha-1(I) chain | CH10_HUMAN | 10 kDa heat shock protein |
| DYHC1_HUMAN | Cytoplasmic dynein 1 heavy chain 1 | CHRD1_HUMAN | CHORD domain-containing protein 1 |
| EF1G_HUMAN | Elongation factor 1-gamma | FKBP3_HUMAN | Peptidyl-prolyl cis-trans isomerase |
| GLSK_HUMAN | Glutaminase kidney isoform, mitochondrial | FKBP4_HUMAN | Peptidyl-prolyl cis-trans isomerase |
| HNRPM_HUMAN | Heterogeneous nuclear ribonucleoprotein M | GBG12_HUMAN | Guanine nucleotide-binding protein G |
| HNRPU_HUMAN | Heterogeneous nuclear ribonucleoprotein U | H12_HUMAN | Histone H1d |
| HORN_HUMAN | Hornerin | H13_HUMAN | Histone H1c |
| HS105_HUMAN | Heat shock protein 105 kDa | H14_HUMAN | Histone H1b |
| IF4G1_HUMAN | eIF-4-gamma 1 | H2B1B_HUMAN | Histone H2B type 1-B |
| IMA2_HUMAN | Importin subunit alpha-2 | H2B1C_HUMAN | Histone H2B type 1-C |
| IPO5_HUMAN | Importin-5 | H2B1D_HUMAN | Histone H2B type 1-D |
| MARE1_HUMAN | End-binding protein 1 | H2B1H_HUMAN | Histone H2B type 1-H |
| NP1L4_HUMAN | Nucleosome assembly protein 1-like 4 | H2B1J_HUMAN | Histone H2B type 2-J |
| PCBP2_HUMAN | Poly(rC)-binding protein 2 | H2B1K_HUMAN | Histone H2B type 2-K |
| PDIA4_HUMAN | Protein disulfide-isomerase A4 | H2B1L_HUMAN | Histone H2B type 2-L |
| PLEC1_HUMAN | Plectin | H2B1M_HUMAN | Histone H2B type 2-M |
| PLST_HUMAN | Plastin-3 | H2B1N_HUMAN | Histone H2B type 2-N |
| PTMA_HUMAN | Prothymosin alpha | H2B1O_HUMAN | Histone H2B type 2-O |
| RHOA_HUMAN | Transforming protein RhoA | H2B2E_HUMAN | Histone H2B type 2-E |
| RINI_HUMAN | Ribonuclease inhibitor | H2B2F_HUMAN | Histone H2B type 2-F |
| ROA2_HUMAN | Heterogeneous nuclear ribonucleoproteins A2/B1 | H2B3B_HUMAN | Histone H2B type 3-B |
| SAHH_HUMAN | Adenosylhomocysteinase | H2BFS_HUMAN | Histone H2B type F-S |
| TBB6_HUMAN | Tubulin beta-6 chain | ITB1_HUMAN | Integrin beta-1 |
| TCPA_HUMAN | TCP-1-alpha | MAP4_HUMAN | Microtubule-associated protein 4 |
| TCPZ_HUMAN | TCP-1-zeta | MDHC_HUMAN | Malate dehydrogenase, cytoplasmic |
| TIF1B_HUMAN | TIF1-beta | NEDD8_HUMAN | NEDD8 |
| TPM2_HUMAN | Tropomyosin beta chain | PDCD5_HUMAN | Programmed cell death protein 5 |
| TRXR1_HUMAN | Thioredoxin reductase 1, cytoplasmic | PEBP1_HUMAN | Phosphatidylethanolamine-binding protein 1 |
| XPO1_HUMAN | Exportin-1 | PSME2_HUMAN | Proteasome activator complex subunit 2 |
|  |  | RAP1B_HUMAN | Ras-related protein Rap-1b |
|  |  | RL12_HUMAN | 60S ribosomal protein L12 |
|  |  | RL30_HUMAN | 60S ribosomal protein L30 |
|  |  | RL9_HUMAN | 60S ribosomal protein L9 |
|  |  | RS10_HUMAN | 40S ribosomal protein S10 |
|  |  | RS17_HUMAN | 40S ribosomal protein S17 |
|  |  | RS18_HUMAN | 40S ribosomal protein S18 |
|  |  | RS19_HUMAN | 40S ribosomal protein S19 |
|  |  | RSSA_HUMAN | 40S ribosomal protein SA |
|  |  | SUMO2_HUMAN | Small ubiquitin-related modifier 2 |
|  |  | SYG_HUMAN | Glycyl-tRNA synthetase |
|  |  | TXND5_HUMAN | Thioredoxin domain-containing protein 5 |
|  |  | VAT1_HUMAN | Synaptic vesicle membrane protein VAT-1 homolog |
|  |  | ZYX_HUMAN | Zyxin |
