# Supplementary material for: Identifying New Therapeutic Targets via Modulation of Protein Corona Formation by Engineered Nanoparticles
Source: PLoS One. 2012 Mar 19;7(3):e33650. doi: 10.1371/journal.pone.0033650 (PMC3307759; doi:10.1371/journal.pone.0033650)
Supplement: Table S2 — Unique proteins present in the corona of +AuNP and −AuNP from OV167 lysate. (DOCX) [file pone.0033650.s005.docx]

**Table S2. Unique proteins present in the corona of ^+^AuNP and ^–^AuNP from OV167 lysate.**

| **Unique to ^+^AuNP** | | **Unique to ^--^AuNP** | |
| --- | --- | --- | --- |
| Gene Name | Full Name | Gene Name | Full name |
| AATM_HUMAN | Fatty acid-binding protein | 1433G_HUMAN | Protein kinase C inhibitor protein 1 |
| ALDOC_HUMAN | Brain-type aldolase | 1433T_HUMAN | 14-3-3 protein theta |
| CH10_HUMAN | 10 kDa heat shock protein | ACLY_HUMAN | ATP-citrate synthase |
| CYBP_HUMAN | Calcyclin-binding protein | ACTN1_HUMAN | Alpha-actinin-1 |
| FUBP1_HUMAN | FUSE-binding protein 1 | ACTN4_HUMAN | Alpha-actinin-4 |
| FUBP2_HUMAN | FUSE-binding protein 2 | ANXA5_HUMAN | Annexin A5 |
| G6PI_HUMAN | Glucose-6-phosphate isomerase | ATPA_HUMAN | ATP synthase subunit alpha, mitochondrial |
| HDGF_HUMAN | Hepatoma-derived growth factor | C1QBP_HUMAN | GC1q-R protein |
| HNRPQ_HUMAN | Heterogeneous nuclear ribonucleoprotein Q | CAP1_HUMAN | Adenylyl cyclase-associated protein 1 |
| LAP2A_HUMAN | Lamina-associated polypeptide 2, | CAZA1_HUMAN | F-actin-capping protein subunit alpha-1 |
| LAP2B_HUMAN | Lamina-associated polypeptide 2, isoforms beta/gamma | CNDP2_HUMAN | Cytosolic non-specific dipeptidase |
| MARE1_HUMAN | End-binding protein 1 | DDX17_HUMAN | DEAD box protein 17 |
| MDHM_HUMAN | Malate dehydrogenase, mitochondrial | DHX9_HUMAN | ATP-dependent RNA helicase A |
| MOES_HUMAN | Moesin | EF1B_HUMAN | EF-1-beta |
| PA2G4_HUMAN | Proliferation-associated protein 2G4 | EF1G_HUMAN | EF-1-gamma |
| PARK7_HUMAN | Protein DJ-1 | ENPL_HUMAN | Endoplasmin |
| PARP1_HUMAN | PARP-1 | ERO1A_HUMAN | ERO1-like protein alpha |
| S10A4_HUMAN | Protein S100-A4 | FERM2_HUMAN | Fermitin family homolog 2 |
|  |  | HNRH1_HUMAN | Heterogeneous nuclear ribonucleoprotein H |
|  |  | HNRPC_HUMAN | Heterogeneous nuclear ribonucleoproteins C1/C2 |
|  |  | HNRPD_HUMAN | Heterogeneous nuclear ribonucleoprotein D0 |
|  |  | HNRPF_HUMAN | Heterogeneous nuclear ribonucleoprotein F |
|  |  | HS90A_HUMAN | Heat shock protein HSP 90-alpha |
|  |  | HYOU1_HUMAN | Hypoxia up-regulated protein 1 |
|  |  | IF2BL_HUMAN | Eukaryotic translation initiation factor 2 subunit 2-like protein |
|  |  | IF2B_HUMAN | Eukaryotic translation initiation factor 2 subunit 2 |
|  |  | ILF3_HUMAN | Interleukin enhancer-binding factor 3 |
|  |  | IMA2_HUMAN | Importin subunit alpha-2 |
|  |  | KU70_HUMAN | KU70 |
|  |  | KU86_HUMAN | TP-dependent DNA helicase 2 subunit 2 |
|  |  | LMNA_HUMAN | Prelamin-A/C |
|  |  | MYL6_HUMAN | Myosin light polypeptide 6 |
|  |  | NASP_HUMAN | Nuclear autoantigenic sperm protein |
|  |  | NONO_HUMAN | 55 kDa nuclear protein |
|  |  | NUDC_HUMAN | Nuclear migration protein nudC |
|  |  | NUDT5_HUMAN | ADP-sugar pyrophosphatase |
|  |  | PGRC1_HUMAN | Membrane-associated progesterone receptor component 1 |
|  |  | PHB2_HUMAN | Prohibitin-2 |
|  |  | PRDX6_HUMAN | Peroxiredoxin-6 |
|  |  | PRS4_HUMAN | 26S protease regulatory subunit 4 |
|  |  | PSA5_HUMAN | Proteasome subunit alpha type-5 |
|  |  | PSD12_HUMAN | 26S proteasome non-ATPase regulatory subunit 12 |
|  |  | PUR9_HUMAN | Bifunctional purine biosynthesis protein |
|  |  | PYRG1_HUMAN | CTP synthase 1 |
|  |  | RAB7A_HUMAN | Ras-related protein Rab-7a |
|  |  | RL15_HUMAN | 60S ribosomal protein L15 |
|  |  | RL5_HUMAN | 60S ribosomal protein L5 |
|  |  | RL6_HUMAN | 60S ribosomal protein L6 |
|  |  | RL7A_HUMAN | 60S ribosomal protein L7a |
|  |  | RPN1_HUMAN | Ribophorin I |
|  |  | RS15_HUMAN | 40S ribosomal protein S15 |
|  |  | RS18_HUMAN | 40S ribosomal protein S18 |
|  |  | RS20_HUMAN | 40S ribosomal protein S20 |
|  |  | RS5_HUMAN | 40S ribosomal protein S5 |
|  |  | RSSA_HUMAN | 40S ribosomal protein SA |
|  |  | SET_HUMAN | Protein SET |
|  |  | SKP1_HUMAN | S-phase kinase-associated protein 1 |
|  |  | TCPA_HUMAN | TCP-1-alpha |
|  |  | TCPD_HUMAN | TCP-1-delta |
|  |  | TCPE_HUMAN | TCP-1-epsilon |
|  |  | TCPQ_HUMAN | TCP-1-theta |
|  |  | TERA_HUMAN | TER ATPase |
|  |  | THOC4_HUMAN | THO complex subunit 4 |
|  |  | TIF1B_HUMAN | TIF1-beta |
|  |  | TPD54_HUMAN | Tumor protein D54 |
|  |  | TPM4_HUMAN | Tropomyosin alpha-4 chain |
|  |  | UAP56_HUMAN | Spliceosome RNA helicase DDX39B |
|  |  | UBE2N_HUMAN | Ubiquitin-conjugating enzyme E2 N |
|  |  | VDAC1_HUMAN | VDAC-1 |
|  |  | XPO2_HUMAN | Exportin-2 |
