# Supplementary material for: Identifying New Therapeutic Targets via Modulation of Protein Corona Formation by Engineered Nanoparticles
Source: PLoS One. 2012 Mar 19;7(3):e33650. doi: 10.1371/journal.pone.0033650 (PMC3307759; doi:10.1371/journal.pone.0033650)
Supplement: Table S3 — All Proteins OSE Lysate. (DOCX) [file pone.0033650.s006.docx]

**Table S3**

| **All Proteins OSE Lysate** | |
| --- | --- |
| Gene Name | Full Name |
| 1433T_HUMAN | 14-3-3 protein theta |
| 1433Z_HUMAN | 14-3-3 protein zeta/delta |
| ACTG_HUMAN | Actin, cytoplasmic 2 |
| ACTN1_HUMAN | Alpha-actinin-1 |
| ACTN4_HUMAN | Alpha-actinin-4 |
| AHNK_HUMAN | Neuroblast differentiation-associated protein |
| ALDOA_HUMAN | Fructose-bisphosphate aldolase A |
| ANXA1_HUMAN | Annexin A1 |
| ANXA2_HUMAN | Annexin A2 |
| ANXA5_HUMAN | Annexin A5 |
| ATPA_HUMAN | ATP synthase subunit alpha, mitochondrial |
| ATPB_HUMAN | ATP synthase subunit beta, mitochondrial |
| BASI_HUMAN | Basigin |
| BASP1_HUMAN | Brain acid soluble protein 1 |
| CALD1_HUMAN | Caldesmon |
| CALR_HUMAN | Calreticulin |
| CALX_HUMAN | Calnexin |
| CAP1_HUMAN | Adenylyl cyclase-associated protein 1 |
| CH60_HUMAN | 60 kDa heat shock protein, mitochondrial |
| CKAP4_HUMAN | Cytoskeleton-associated protein 4 |
| CLH1_HUMAN | Clathrin heavy chain 1 |
| COF1_HUMAN | Cofilin-1 |
| CPNS1_HUMAN | Calpain small subunit 1 |
| EF1A1_HUMAN | Elongation factor 1-alpha 1 |
| EF1A3_HUMAN | Putative elongation factor 1-alpha-like 3 |
| EF2_HUMAN | Elongation factor 2 |
| ENOA_HUMAN | Alpha-enolase |
| ENPL_HUMAN | Endoplasmin |
| EZRI_HUMAN | Ezrin |
| FAS_HUMAN | Fatty acid synthase |
| FLNA_HUMAN | Filamin-A |
| FLNB_HUMAN | Filamin-B |
| FLNC_HUMAN | Filamin-C |
| G3P_HUMAN | Glyceraldehyde-3-phosphate dehydrogenase |
| GANAB_HUMAN | Neutral alpha-glucosidase AB |
| GDIR1_HUMAN | Rho GDP-dissociation inhibitor 1 |
| GLU2B_HUMAN | Glucosidase 2 subunit beta |
| GRP75_HUMAN | Stress-70 protein, mitochondrial |
| GRP78_HUMAN | 78 kDa glucose-regulated protein |
| GSTP1_HUMAN | Glutathione S-transferase P |
| HMGB1_HUMAN | High mobility group protein B1 |
| HNRPK_HUMAN | Heterogeneous nuclear ribonucleoprotein K |
| HSP71_HUMAN | Heat shock 70 kDa protein 1A/1B |
| HSP74_HUMAN | Heat shock 70 kDa protein 4 |
| HSP7C_HUMAN | Heat shock cognate 71 kDa protein |
| IF4A1_HUMAN | Eukaryotic initiation factor 4A-I |
| IF5A1_HUMAN | Eukaryotic translation initiation factor 5A-1 |
| IMB1_HUMAN | Importin subunit beta-1 |
| IQGA1_HUMAN | Ras GTPase-activating-like protein |
| K1C10_HUMAN | Keratin, type I cytoskeletal 10 |
| K1C18_HUMAN | Keratin, type I cytoskeletal 18 |
| K1C19_HUMAN | Keratin, type I cytoskeletal 19 |
| K1C9_HUMAN | Keratin, type I cytoskeletal 9 |
| K2C1_HUMAN | Keratin, type II cytoskeletal 1 |
| K2C7_HUMAN | Keratin, type II cytoskeletal 7 |
| K2C8_HUMAN | Keratin, type II cytoskeletal 8 |
| KPYM_HUMAN | Pyruvate kinase isozymes M1/M2 |
| LDHA_HUMAN | L-lactate dehydrogenase A chain |
| LDHB_HUMAN | L-lactate dehydrogenase B chain |
| LEG1_HUMAN | Galectin-1 |
| LMNA_HUMAN | Prelamin-A/C |
| MDHM_HUMAN | Malate dehydrogenase, mitochondrial |
| MOES_HUMAN | Moesin |
| MYH9_HUMAN | Myosin-9 |
| MYL6_HUMAN | Myosin light polypeptide 6 |
| NACA_HUMAN | Nascent polypeptide-associated complex subunit alpha |
| NDKB_HUMAN | Nucleoside diphosphate kinase B |
| NPM_HUMAN | Nucleophosmin |
| NUCL_HUMAN | Nucleolin |
| PDIA1_HUMAN | Protein disulfide-isomerase |
| PDIA3_HUMAN | Protein disulfide-isomerase A3 |
| PDIA6_HUMAN | Protein disulfide-isomerase A6 |
| PGK1_HUMAN | Phosphoglycerate kinase 1 |
| PPIA_HUMAN | Peptidyl-prolyl cis-trans isomerase A |
| PPIB_HUMAN | Peptidyl-prolyl cis-trans isomerase B |
| PRDX1_HUMAN | Peroxiredoxin-1 |
| PRDX6_HUMAN | Peroxiredoxin-6 |
| PRKDC_HUMAN | DNA-dependent protein kinase catalytic subunit |
| PROF1_HUMAN | Profilin-1 |
| PTRF_HUMAN | Polymerase I and transcript release factor |
| RAB1B_HUMAN | Ras-related protein Rab-1B |
| RL3_HUMAN | 60S ribosomal protein L3 |
| RLA2_HUMAN | 60S acidic ribosomal protein P2 |
| RS15_HUMAN | 40S ribosomal protein S15 |
| RS20_HUMAN | 40S ribosomal protein S20 |
| RS3_HUMAN | 40S ribosomal protein S3 |
| SEPT2_HUMAN | Septin-2 |
| SERPH_HUMAN | Serpin H1 |
| STIP1_HUMAN | Stress-induced-phosphoprotein 1 |
| TAGL2_HUMAN | Transgelin-2 |
| TBA1A_HUMAN | Alpha-tubulin 3 |
| TBB2C_HUMAN | Tubulin beta-2C chain |
| TBB5_HUMAN | Tubulin beta chain |
| TCPB_HUMAN | T-complex protein 1 subunit beta |
| TCPD_HUMAN | T-complex protein 1 subunit delta |
| TCPE_HUMAN | T-complex protein 1 subunit epsilon |
| TCPG_HUMAN | T-complex protein 1 subunit gamma |
| TCPH_HUMAN | T-complex protein 1 subunit eta |
| TERA_HUMAN | Transitional endoplasmic reticulum ATPase |
| THIL_HUMAN | Acetyl-CoA acetyltransferase, mitochondrial |
| TKT_HUMAN | Transketolase |
| TLN1_HUMAN | Talin-1 |
| TMSL3_HUMAN | Thymosin beta-4-like protein 3 |
| TPD54_HUMAN | Tumor protein D54 |
| TPIS_HUMAN | Triosephosphate isomerase |
| TPM4_HUMAN | Tropomyosin alpha-4 chain |
| TYB4_HUMAN | Thymosin beta-4 |
| UBA1_HUMAN | Ubiquitin-like modifier-activating enzyme 1 |
| UCHL1_HUMAN | Ubiquitin carboxyl-terminal hydrolase isozyme L1 |
| VIME_HUMAN | Vimentin |
| XPO2_HUMAN | Exportin-2 |
| YBOX1_HUMAN | Nuclease-sensitive element-binding protein 1 |
