# Supplementary material for: Identifying New Therapeutic Targets via Modulation of Protein Corona Formation by Engineered Nanoparticles
Source: PLoS One. 2012 Mar 19;7(3):e33650. doi: 10.1371/journal.pone.0033650 (PMC3307759; doi:10.1371/journal.pone.0033650)
Supplement: Table S4 — All proteins present in the corona of +AuNP from OSE lysates. (DOCX) [file pone.0033650.s007.docx]

**Table S4: All proteins present in the corona of ^+^AuNP from OSE lysates.**

| **All Proteins in OSE ^+^AuNP corona** | |
| --- | --- |
| Gene Name | Full Name |
| 1433B_HUMAN | Protein kinase C inhibitor protein 1 |
| 1433T_HUMAN | 14-3-3 protein tau |
| 1433Z_HUMAN | 14-3-3 protein zeta/delta |
| ACTG_HUMAN | Gamma-actin |
| ACTN1_HUMAN | Alpha-actinin-1 |
| ACTN4_HUMAN | Alpha-actinin-4 |
| AHNK_HUMAN | Neuroblast differentiation-associated protein |
| ALDOA_HUMAN | Fructose-bisphosphate aldolase A |
| ANXA1_HUMAN | Annexin A1 |
| ANXA2_HUMAN | Annexin A2 |
| ANXA5_HUMAN | Annexin A5 |
| AT1A1_HUMAN | Sodium/potassium-transporting ATPase subunit alpha-1 |
| ATPA_HUMAN | ATP synthase subunit alpha, mitochondrial |
| ATPB_HUMAN | ATP synthase subunit beta, mitochondrial |
| C1QBP_HUMAN | GC1q-R protein |
| CALD1_HUMAN | Caldesmon |
| CALR_HUMAN | Calreticulin |
| CALU_HUMAN | Calumenin |
| CALX_HUMAN | Calnexin |
| CAP1_HUMAN | Adenylyl cyclase-associated protein 1 |
| CAPR1_HUMAN | Caprin-1 |
| CH60_HUMAN | 60 kDa heat shock protein, mitochondrial |
| CKAP4_HUMAN | Cytoskeleton-associated protein 4 |
| CLH1_HUMAN | Clathrin heavy chain 1 |
| CO1A1_HUMAN | Collagen alpha-1(I) chain |
| COF1_HUMAN | Cofilin-1 |
| DYHC1_HUMAN | Cytoplasmic dynein 1 heavy chain 1 |
| EF1A1_HUMAN | EF-1-alpha-1 |
| EF1A3_HUMAN | EF-1-alpha-like 3 |
| EF1G_HUMAN | EF-1-gamma |
| EF2_HUMAN | Elongation factor 2 |
| ENOA_HUMAN | Alpha-enolase |
| ENPL_HUMAN | Endoplasmin |
| EZRI_HUMAN | Ezrin |
| FAS_HUMAN | Fatty acid synthase |
| FLNA_HUMAN | Filamin-A |
| FLNB_HUMAN | Filamin-B |
| FLNC_HUMAN | Filamin-C |
| G3P_HUMAN | GAPDH |
| GANAB_HUMAN | Neutral alpha-glucosidase AB |
| GLSK_HUMAN | Glutaminase kidney isoform, mitochondrial |
| GLU2B_HUMAN | Glucosidase 2 subunit beta |
| GRP75_HUMAN | Stress-70 protein, mitochondrial |
| GRP78_HUMAN | 78 kDa glucose-regulated protein |
| HNRPK_HUMAN | Heterogeneous nuclear ribonucleoprotein K |
| HNRPM_HUMAN | Heterogeneous nuclear ribonucleoprotein M |
| HNRPU_HUMAN | Heterogeneous nuclear ribonucleoprotein U |
| HORN_HUMAN | Hornerin |
| HS105_HUMAN | Heat shock protein 105 kDa |
| HSP71_HUMAN | Heat shock 70 kDa protein 1A/1B |
| HSP7C_HUMAN | Heat shock cognate 71 kDa protein |
| HSPB1_HUMAN | Heat shock protein beta-1 |
| IF4A1_HUMAN | Eukaryotic initiation factor 4A-I |
| IF4G1_HUMAN | eIF-4-gamma 1 |
| IMA2_HUMAN | Importin subunit alpha-2 |
| IMB1_HUMAN | Importin subunit beta-1 |
| IPO5_HUMAN | Importin-5 |
| IQGA1_HUMAN | Ras GTPase-activating-like protein |
| K1C10_HUMAN | Cytokeratin-10 |
| K1C18_HUMAN | Cytokeratin-18 |
| K1C19_HUMAN | Cytokeratin-19 |
| K1C9_HUMAN | Cytokeratin-9 |
| K22E_HUMAN | Cytokeratin-2e |
| K2C1_HUMAN | Cytokeratin-1 |
| K2C7_HUMAN | Cytokeratin-7 |
| K2C8_HUMAN | Cytokeratin-8 |
| KPYM_HUMAN | Pyruvate kinase isozymes M1/M2 |
| LDHA_HUMAN | LDH-A |
| LDHB_HUMAN | LDH-B |
| LEG1_HUMAN | Galectin-1 |
| LMNA_HUMAN | Prelamin-A/C |
| MARCS_HUMAN | MARCKS |
| MARE1_HUMAN | End-binding protein 1 |
| MOES_HUMAN | Moesin |
| MYH9_HUMAN | Myosin-9 |
| MYL6_HUMAN | Myosin light polypeptide 6 |
| NDKB_HUMAN | Nucleoside diphosphate kinase B |
| NP1L4_HUMAN | Nucleosome assembly protein 1-like 4 |
| NPM_HUMAN | Nucleophosmin |
| NUCL_HUMAN | Nucleolin |
| PCBP2_HUMAN | Poly(rC)-binding protein 2 |
| PDIA1_HUMAN | Protein disulfide-isomerase |
| PDIA3_HUMAN | Protein disulfide-isomerase A3 |
| PDIA4_HUMAN | Protein disulfide-isomerase A4 |
| PGK1_HUMAN | Phosphoglycerate kinase 1 |
| PLEC1_HUMAN | Plectin |
| PLST_HUMAN | Plastin-3 |
| PPIA_HUMAN | Peptidyl-prolyl cis-trans isomerase A |
| PRDX1_HUMAN | Peroxiredoxin-1 |
| PRKDC_HUMAN | DNA-dependent protein kinase catalytic subunit |
| PTMA_HUMAN | Prothymosin alpha |
| PTRF_HUMAN | Polymerase I and transcript release factor |
| RHOA_HUMAN | Transforming protein RhoA |
| RINI_HUMAN | Ribonuclease inhibitor |
| RLA2_HUMAN | 60S acidic ribosomal protein P2 |
| ROA2_HUMAN | Heterogeneous nuclear ribonucleoproteins A2/B1 |
| SAHH_HUMAN | Adenosylhomocysteinase |
| SERPH_HUMAN | Serpin H1 |
| SET_HUMAN | Protein SET |
| TAGL2_HUMAN | Transgelin-2 |
| TAGL_HUMAN | Transgelin |
| TBA1A_HUMAN | Tubulin alpha-1A chain |
| TBB2C_HUMAN | Tubulin beta-2C chain |
| TBB5_HUMAN | Tubulin beta chain |
| TBB6_HUMAN | Tubulin beta-6 chain |
| TCPA_HUMAN | T-complex protein 1 subunit alpha |
| TCPB_HUMAN | T-complex protein 1 subunit beta |
| TCPD_HUMAN | T-complex protein 1 subunit delta |
| TCPE_HUMAN | T-complex protein 1 subunit epsilon |
| TCPG_HUMAN | T-complex protein 1 subunit gamma |
| TCPH_HUMAN | T-complex protein 1 subunit eta |
| TCPQ_HUMAN | T-complex protein 1 subunit theta |
| TCPZ_HUMAN | T-complex protein 1 subunit zeta |
| TCTP_HUMAN | Translationally-controlled tumor protein |
| TERA_HUMAN | Transitional endoplasmic reticulum ATPase |
| TIF1B_HUMAN | Transcription intermediary factor 1-beta |
| TKT_HUMAN | Transketolase |
| TLN1_HUMAN | Talin-1 |
| TPIS_HUMAN | Triosephosphate isomerase |
| TPM2_HUMAN | Tropomyosin beta chain |
| TPM4_HUMAN | Tropomyosin alpha-4 chain |
| TRXR1_HUMAN | Thioredoxin reductase 1, cytoplasmic |
| UBA1_HUMAN | Ubiquitin-like modifier-activating enzyme 1 |
| VIME_HUMAN | Vimentin |
| XPO1_HUMAN | Exportin-1 |
| XPO2_HUMAN | Exportin-2 |
