# Supplementary material for: Identifying New Therapeutic Targets via Modulation of Protein Corona Formation by Engineered Nanoparticles
Source: PLoS One. 2012 Mar 19;7(3):e33650. doi: 10.1371/journal.pone.0033650 (PMC3307759; doi:10.1371/journal.pone.0033650)
Supplement: Table S5 — All proteins present in the corona of −AuNP from OSE lysates. (DOCX) [file pone.0033650.s008.docx]

**Table S5: All proteins present in the corona of ^-^AuNP from OSE lysates.**

| **All proteins in the OSE- ^-^AuNP corona** | |
| --- | --- |
| 1433B_HUMAN | 14-3-3 protein beta/alpha |
| 1433G_HUMAN | 14-3-3 protein gamma |
| 1433Z_HUMAN | 14-3-3 protein zeta/delta |
| ACTG_HUMAN | Gamma-actin |
| ACTN1_HUMAN | Alpha-actinin-1 |
| ACTN4_HUMAN | Alpha-actinin-4 |
| AHNK_HUMAN | Neuroblast differentiation-associated protein |
| ALDOA_HUMAN | Fructose-bisphosphate aldolase A |
| ANXA1_HUMAN | Annexin A1 |
| ANXA2_HUMAN | Annexin A2 |
| ANXA5_HUMAN | Annexin A5 |
| ATPA_HUMAN | ATP synthase subunit alpha, mitochondrial |
| ATPO_HUMAN | ATP synthase subunit O, mitochondrial |
| BASP1_HUMAN | Brain acid soluble protein 1 |
| CALR_HUMAN | Calreticulin |
| CALX_HUMAN | Calnexin |
| CAV1_HUMAN | Caveolin-1 |
| CD44_HUMAN | CD44 |
| CH10_HUMAN | 10 kDa heat shock protein, mitochondrial |
| CH60_HUMAN | 60 kDa heat shock protein, mitochondrial |
| CHRD1_HUMAN | Cysteine and histidine-rich domain-containing protein 1 |
| COF1_HUMAN | Cofilin-1 |
| CPNS1_HUMAN | Calpain small subunit 1 |
| EF1A1_HUMAN | EF-1-alpha-1 |
| EF1A3_HUMAN | EF-1-alpha-like 3 |
| ENOA_HUMAN | Alpha-enolase |
| ENPL_HUMAN | Endoplasmin |
| EZRI_HUMAN | Ezrin |
| FKBP3_HUMAN | Peptidyl-prolyl cis-trans isomerase FKBP3 |
| FKBP4_HUMAN | Peptidyl-prolyl cis-trans isomerase FKBP4 |
| FLNA_HUMAN | Filamin-A |
| G3P_HUMAN | GAPDH |
| GBG12_HUMAN | Guanine nucleotide-binding protein G |
| GRP78_HUMAN | 78 kDa glucose-regulated protein |
| H12_HUMAN | Histone H1d |
| H13_HUMAN | Histone H1c |
| H14_HUMAN | Histone H1b |
| H2B1B_HUMAN | Histone H2B type 1-B |
| H2B1C_HUMAN | Histone H2B type 1-C |
| H2B1D_HUMAN | Histone H2B type 1-D |
| H2B1H_HUMAN | Histone H2B type 1-H |
| H2B1J_HUMAN | Histone H2B type 1-J |
| H2B1K_HUMAN | Histone H2B type 1-K |
| H2B1L_HUMAN | Histone H2B type 1-L |
| H2B1M_HUMAN | Histone H2B type 1-M |
| H2B1N_HUMAN | Histone H2B type 1-N |
| H2B1O_HUMAN | Histone H2B type 1-O |
| H2B2E_HUMAN | Histone H2B type 2-E |
| H2B2F_HUMAN | Histone H2B type 2-F |
| H2B3B_HUMAN | Histone H2B type 3-B |
| H2BFS_HUMAN | Histone H2B type F-S |
| HMGB1_HUMAN | High mobility group protein B1 |
| HNRPK_HUMAN | Heterogeneous nuclear ribonucleoprotein K |
| HSP7C_HUMAN | Heat shock cognate 71 kDa protein |
| HSPB1_HUMAN | Heat shock protein beta-1 |
| IQGA1_HUMAN | Ras GTPase-activating-like protein |
| ITB1_HUMAN | Integrin beta-1 |
| K1C10_HUMAN | Keratin, type I cytoskeletal 10 |
| K1C18_HUMAN | Keratin, type I cytoskeletal 18 |
| K1C19_HUMAN | Keratin, type I cytoskeletal 19 |
| K1C9_HUMAN | Keratin, type I cytoskeletal 9 |
| K22E_HUMAN | Keratin, type II cytoskeletal 2 epidermal |
| K2C1_HUMAN | Keratin, type II cytoskeletal 1 |
| K2C8_HUMAN | Keratin, type II cytoskeletal 8 |
| KPYM_HUMAN | Pyruvate kinase isozymes M1/M2 |
| LDHA_HUMAN | L-lactate dehydrogenase A chain |
| LDHB_HUMAN | L-lactate dehydrogenase B chain |
| LEG1_HUMAN | Galectin-1 |
| MAP4_HUMAN | Microtubule-associated protein 4 |
| MARCS_HUMAN | Myristoylated alanine-rich C-kinase substrate |
| MDHC_HUMAN | Malate dehydrogenase, cytoplasmic |
| MYH9_HUMAN | Myosin-9 |
| MYL6_HUMAN | Myosin light polypeptide 6 |
| NEDD8_HUMAN | NEDD8 |
| PDCD5_HUMAN | Programmed cell death protein 5 |
| PDIA1_HUMAN | Protein disulfide-isomerase |
| PDIA6_HUMAN | Protein disulfide-isomerase A6 |
| PEBP1_HUMAN | Phosphatidylethanolamine-binding protein 1 |
| PGK1_HUMAN | Phosphoglycerate kinase 1 |
| PPIA_HUMAN | Peptidyl-prolyl cis-trans isomerase A |
| PRDX1_HUMAN | Peroxiredoxin-1 |
| PROF1_HUMAN | Profilin-1 |
| PSME2_HUMAN | Proteasome activator complex subunit 2 |
| RAP1B_HUMAN | Ras-related protein Rap-1b |
| RL12_HUMAN | 60S ribosomal protein L12 |
| RL30_HUMAN | 60S ribosomal protein L30 |
| RL9_HUMAN | 60S ribosomal protein L9 |
| RLA2_HUMAN | 60S acidic ribosomal protein P2 |
| RS10_HUMAN | 40S ribosomal protein S10 |
| RS17_HUMAN | 40S ribosomal protein S17 |
| RS18_HUMAN | 40S ribosomal protein S18 |
| RS19_HUMAN | 40S ribosomal protein S19 |
| RS20_HUMAN | 40S ribosomal protein S20 |
| RSSA_HUMAN | 40S ribosomal protein SA |
| SERPH_HUMAN | Serpin H1 |
| SET_HUMAN | Protein SET |
| SUMO2_HUMAN | Small ubiquitin-related modifier 2 |
| SYG_HUMAN | Glycyl-tRNA synthetase |
| TAGL2_HUMAN | Transgelin-2 |
| TAGL_HUMAN | Transgelin |
| TBA1A_HUMAN | Tubulin alpha-1A chain |
| TBB2C_HUMAN | Tubulin beta-2C chain |
| TBB5_HUMAN | Tubulin beta chain |
| TCPQ_HUMAN | TCP-1-theta |
| TCTP_HUMAN | Translationally-controlled tumor protein |
| TERA_HUMAN | Transitional endoplasmic reticulum ATPase |
| TMSL3_HUMAN | Thymosin beta-4-like protein 3 |
| TPD54_HUMAN | Tumor protein D54 |
| TPIS_HUMAN | Triosephosphate isomerase |
| TXND5_HUMAN | Thioredoxin domain-containing protein 5 |
| TYB4_HUMAN | Thymosin beta-4 |
| VAT1_HUMAN | Synaptic vesicle membrane protein VAT-1 homolog |
| YBOX1_HUMAN | Nuclease-sensitive element-binding protein 1 |
| ZYX_HUMAN | Zyxin |
