# Supplementary material for: Identifying New Therapeutic Targets via Modulation of Protein Corona Formation by Engineered Nanoparticles
Source: PLoS One. 2012 Mar 19;7(3):e33650. doi: 10.1371/journal.pone.0033650 (PMC3307759; doi:10.1371/journal.pone.0033650)
Supplement: Table S6 — All proteins present in OV167 lysate. (DOCX) [file pone.0033650.s009.docx]

**Table S6: All proteins present in OV167 lysate.**

| **All proteins in OV167 lysate** | |
| --- | --- |
| Gene Name | Full Name |
| 1433E_HUMAN | 14-3-3 protein epsilon |
| 1433Z_HUMAN | 14-3-3 protein zeta/delta |
| 6PGD_HUMAN | 6-phosphogluconate dehydrogenase, decarboxylating |
| ACTB_HUMAN | Actin, cytoplasmic 1 |
| ACTG_HUMAN | Actin, cytoplasmic 2 |
| ADT2_HUMAN | ADP/ATP translocase 2 |
| AHNK_HUMAN | Neuroblast differentiation-associated protein |
| ALDOA_HUMAN | Fructose-bisphosphate aldolase A |
| ANXA6_HUMAN | Annexin A6 |
| ARF1_HUMAN | ADP-ribosylation factor 1 |
| ARF3_HUMAN | ADP-ribosylation factor 3 |
| CALD1_HUMAN | Caldesmon |
| CALM_HUMAN | Calmodulin |
| CALR_HUMAN | Calreticulin |
| CALX_HUMAN | Calnexin |
| CAPZB_HUMAN | F-actin-capping protein subunit beta |
| CH60_HUMAN | 60 kDa heat shock protein, mitochondrial |
| CLH1_HUMAN | Clathrin heavy chain 1 |
| CLIC1_HUMAN | Chloride intracellular channel protein 1 |
| COF1_HUMAN | Cofilin-1 |
| EF1A1_HUMAN | Elongation factor 1-alpha 1 |
| EF2_HUMAN | Elongation factor 2 |
| EFTU_HUMAN | Elongation factor Tu, mitochondrial |
| ENOA_HUMAN | Alpha-enolase |
| EZRI_HUMAN | Ezrin |
| FAS_HUMAN | Fatty acid synthase |
| FLNA_HUMAN | Filamin-A |
| G3P_HUMAN | Glyceraldehyde-3-phosphate dehydrogenase |
| GANAB_HUMAN | Neutral alpha-glucosidase AB |
| GBLP_HUMAN | Guanine nucleotide-binding protein subunit beta-2-like 1 |
| GDIB_HUMAN | Rab GDP dissociation inhibitor beta |
| GLU2B_HUMAN | Glucosidase 2 subunit beta |
| GRP75_HUMAN | Stress-70 protein, mitochondrial |
| GRP78_HUMAN | 78 kDa glucose-regulated protein |
| GSTP1_HUMAN | Glutathione S-transferase P |
| H2A1C_HUMAN | Histone H2A type 1-C |
| H2A1D_HUMAN | Histone H2A type 1-D |
| H2A1H_HUMAN | Histone H2A type 1-H |
| H2A1J_HUMAN | Histone H2A type 1-J |
| H2A1_HUMAN | Histone H2A/p |
| H2A3_HUMAN | Histone H2A type 3 |
| H2AJ_HUMAN | Histone H2A.J |
| HMGB1_HUMAN | High mobility group protein B1 |
| HNRPK_HUMAN | Heterogeneous nuclear ribonucleoprotein K |
| HNRPU_HUMAN | Heterogeneous nuclear ribonucleoprotein U |
| HSP7C_HUMAN | Heat shock cognate 71 kDa protein |
| IF4A1_HUMAN | Eukaryotic initiation factor 4A-I |
| IF5A1_HUMAN | Eukaryotic translation initiation factor 5A-1 |
| KPYM_HUMAN | Pyruvate kinase isozymes M1/M2 |
| LA_HUMAN | Lupus La protein |
| LDHA_HUMAN | L-lactate dehydrogenase A chain |
| LEG1_HUMAN | Galectin-1 |
| LPPRC_HUMAN | Leucine-rich PPR motif-containing protein, mitochondrial |
| MATR3_HUMAN | Matrin-3 |
| MYH9_HUMAN | Myosin-9 |
| NEST_HUMAN | Nestin |
| NQO1_HUMAN | NAD(P)H dehydrogenase [quinone] 1 |
| NUCKS_HUMAN | Nuclear ubiquitous casein and cyclin-dependent kinases substrate |
| PAIRB_HUMAN | Plasminogen activator inhibitor 1 RNA-binding protein |
| PCBP1_HUMAN | Poly(rC)-binding protein 1 |
| PDIA1_HUMAN | Protein disulfide-isomerase |
| PDIA4_HUMAN | Protein disulfide-isomerase A4 |
| PDIA6_HUMAN | Protein disulfide-isomerase A6 |
| PPIA_HUMAN | Peptidyl-prolyl cis-trans isomerase A |
| PPIB_HUMAN | Peptidyl-prolyl cis-trans isomerase B |
| PRDX1_HUMAN | Peroxiredoxin-1 |
| PROF1_HUMAN | Profilin-1 |
| PTBP1_HUMAN | Polypyrimidine tract-binding protein 1 |
| PTMA_HUMAN | Prothymosin alpha |
| RL4_HUMAN | 60S ribosomal protein L4 |
| RLA0_HUMAN | 60S acidic ribosomal protein P0 |
| ROA1_HUMAN | Heterogeneous nuclear ribonucleoprotein A1 |
| ROA2_HUMAN | Heterogeneous nuclear ribonucleoproteins A2/B1 |
| RPN2_HUMAN | Dolichyl-diphosphooligosaccharide--protein glycosyltransferase subunit 2 |
| RS17_HUMAN | 40S ribosomal protein S17 |
| RS19_HUMAN | 40S ribosomal protein S19 |
| RS2_HUMAN | 40S ribosomal protein S2 |
| RS3_HUMAN | 40S ribosomal protein S3 |
| RS4X_HUMAN | 40S ribosomal protein S4, X isoform |
| RSU1_HUMAN | Ras suppressor protein 1 |
| SAHH_HUMAN | Adenosylhomocysteinase |
| SERPH_HUMAN | Serpin H1 |
| SFPQ_HUMAN | Splicing factor, proline- and glutamine-rich |
| STIP1_HUMAN | Stress-induced-phosphoprotein 1 |
| TAGL_HUMAN | Transgelin |
| TBA1B_HUMAN | Tubulin alpha-1B chain |
| TBB5_HUMAN | Tubulin beta chain |
| TCPG_HUMAN | T-complex protein 1 subunit gamma |
| TCPH_HUMAN | T-complex protein 1 subunit eta |
| TCPZ_HUMAN | T-complex protein 1 subunit zeta |
| TEBP_HUMAN | Prostaglandin E synthase 3 |
| TMED2_HUMAN | Transmembrane emp24 domain-containing protein 2 |
| TPM2_HUMAN | Tropomyosin beta chain |
| UBA1_HUMAN | Ubiquitin-like modifier-activating enzyme 1 |
| VASP_HUMAN | Vasodilator-stimulated phosphoprotein |
| VIME_HUMAN | Vimentin |
| VINC_HUMAN | Vinculin |
| YBOX1_HUMAN | Nuclease-sensitive element-binding protein 1 |
