# Supplementary material for: Identifying New Therapeutic Targets via Modulation of Protein Corona Formation by Engineered Nanoparticles
Source: PLoS One. 2012 Mar 19;7(3):e33650. doi: 10.1371/journal.pone.0033650 (PMC3307759; doi:10.1371/journal.pone.0033650)
Supplement: Table S7 — All proteins in the OV167 +AuNP corona. (DOCX) [file pone.0033650.s010.docx]

**Table S7: All proteins in the OV167 ^+^AuNP corona.**

| **All proteins in the OV167 ^+^AuNP corona** | |
| --- | --- |
| 1433B_HUMAN | 14-3-3 protein beta/alpha |
| AATM_HUMAN | Aspartate aminotransferase, mitochondrial |
| ACTB_HUMAN | Actin, cytoplasmic 1 |
| ACTG_HUMAN | Gamma-actin |
| AHNK_HUMAN | Neuroblast differentiation-associated protein |
| ALDOA_HUMAN | Fructose-bisphosphate aldolase A |
| ALDOC_HUMAN | Fructose-bisphosphate aldolase C |
| ANXA2_HUMAN | Annexin A2 |
| CALD1_HUMAN | Caldesmon |
| CH10_HUMAN | 10 kDa heat shock protein, mitochondrial |
| CH60_HUMAN | 60 kDa heat shock protein, mitochondrial |
| COF1_HUMAN | Cofilin-1 |
| CYBP_HUMAN | Calcyclin-binding protein |
| ECHA_HUMAN | Trifunctional enzyme subunit alpha, mitochondrial |
| EF2_HUMAN | Elongation factor 2 |
| ENOA_HUMAN | Alpha-enolase |
| EZRI_HUMAN | Ezrin |
| FLNA_HUMAN | Filamin-A |
| FUBP1_HUMAN | Far upstream element-binding protein 1 |
| FUBP2_HUMAN | Far upstream element-binding protein 2 |
| G3P_HUMAN | GAPDH |
| G6PI_HUMAN | Glucose-6-phosphate isomerase |
| GDIB_HUMAN | Rab GDP dissociation inhibitor beta |
| GRP75_HUMAN | Stress-70 protein, mitochondrial |
| GRP78_HUMAN | 78 kDa glucose-regulated protein |
| HDGF_HUMAN | Hepatoma-derived growth factor |
| HNRPK_HUMAN | Heterogeneous nuclear ribonucleoprotein K |
| HNRPM_HUMAN | Heterogeneous nuclear ribonucleoprotein M |
| HNRPQ_HUMAN | Heterogeneous nuclear ribonucleoprotein Q |
| HNRPU_HUMAN | Heterogeneous nuclear ribonucleoprotein U |
| HSP74_HUMAN | Heat shock 70 kDa protein 4 |
| HSP7C_HUMAN | Heat shock cognate 71 kDa protein |
| IF5A1_HUMAN | Eukaryotic translation initiation factor 5A-1 |
| KPYM_HUMAN | Pyruvate kinase isozymes M1/M2 |
| LAP2A_HUMAN | Lamina-associated polypeptide 2, isoform alpha |
| LAP2B_HUMAN | Lamina-associated polypeptide 2, isoforms beta/gamma |
| LDHA_HUMAN | L-lactate dehydrogenase A chain |
| LDHB_HUMAN | L-lactate dehydrogenase B chain |
| MARE1_HUMAN | Microtubule-associated protein RP/EB family member 1 |
| MDHM_HUMAN | Malate dehydrogenase, mitochondrial |
| MOES_HUMAN | Moesin |
| NACA_HUMAN | Nascent polypeptide-associated complex subunit alpha |
| NDKB_HUMAN | Nucleoside diphosphate kinase B |
| NEST_HUMAN | Nestin |
| NPM_HUMAN | Nucleophosmin |
| NQO1_HUMAN | NQO1_HUMAN |
| NUCKS_HUMAN | Nuclear ubiquitous casein and cyclin-dependent kinases substrate |
| NUCL_HUMAN | Nucleolin |
| PA2G4_HUMAN | Proliferation-associated protein 2G4 |
| PAIRB_HUMAN | Plasminogen activator inhibitor 1 RNA-binding protein |
| PARK7_HUMAN | Protein DJ-1 |
| PARP1_HUMAN | Poly [ADP-ribose] polymerase 1 |
| PCBP1_HUMAN | Poly(rC)-binding protein 1 |
| PDIA1_HUMAN | Protein disulfide-isomerase |
| PDIA3_HUMAN | Protein disulfide-isomerase A3 |
| PDIA6_HUMAN | Protein disulfide-isomerase A6 |
| PGAM1_HUMAN | Phosphoglycerate mutase 1 |
| PGK1_HUMAN | Phosphoglycerate kinase 1 |
| PRDX1_HUMAN | Peroxiredoxin-1 |
| PROF1_HUMAN | Profilin-1 |
| PTBP1_HUMAN | Polypyrimidine tract-binding protein 1 |
| PTMA_HUMAN | Prothymosin alpha |
| RLA2_HUMAN | 60S acidic ribosomal protein P2 |
| ROA2_HUMAN | Heterogeneous nuclear ribonucleoproteins A2/B1 |
| RS3A_HUMAN | 40S ribosomal protein S3a |
| RSU1_HUMAN | Ras suppressor protein 1 |
| S10A4_HUMAN | Protein S100-A4 |
| SERPH_HUMAN | Serpin H1 |
| STIP1_HUMAN | Stress-induced-phosphoprotein 1 |
| TAGL_HUMAN | Transgelin |
| TBA1B_HUMAN | Tubulin alpha-1B chain |
| TBB5_HUMAN | Tubulin beta chain |
| TCPB_HUMAN | TCP-1-beta |
| TKT_HUMAN | Transketolase |
| TPIS_HUMAN | Triosephosphate isomerase |
| VIME_HUMAN | Vimentin |
| VINC_HUMAN | Vinculin |
| YBOX1_HUMAN | Nuclease-sensitive element-binding protein 1 |
