# Supplementary material for: Identifying New Therapeutic Targets via Modulation of Protein Corona Formation by Engineered Nanoparticles
Source: PLoS One. 2012 Mar 19;7(3):e33650. doi: 10.1371/journal.pone.0033650 (PMC3307759; doi:10.1371/journal.pone.0033650)
Supplement: Table S8 — All proteins in the OV167 −AuNP corona. (DOCX) [file pone.0033650.s011.docx]

**Table S8: All proteins in the OV167 ^–^AuNP corona.**

| **All proteins in the OV167 ^–^AuNP corona** | |
| --- | --- |
| 1433B_HUMAN | 14-3-3 protein beta/alpha |
| 1433E_HUMAN | 14-3-3 protein epsilon |
| 1433G_HUMAN | 14-3-3 protein gamma |
| 1433T_HUMAN | 14-3-3 protein theta |
| 6PGD_HUMAN | 6-phosphogluconate dehydrogenase, decarboxylating |
| ACLY_HUMAN | ATP-citrate synthase |
| ACTB_HUMAN | Actin, cytoplasmic 1 |
| ACTG_HUMAN | Actin, cytoplasmic 2 |
| ACTN1_HUMAN | Alpha-actinin-1 |
| ACTN4_HUMAN | Alpha-actinin-4 |
| AHNK_HUMAN | Neuroblast differentiation-associated protein |
| ALDOA_HUMAN | Fructose-bisphosphate aldolase A |
| ANXA2_HUMAN | Annexin A2 |
| ANXA5_HUMAN | Annexin A5 |
| ANXA6_HUMAN | Annexin A6 |
| ARF1_HUMAN | ADP-ribosylation factor 1 |
| ARF3_HUMAN | ADP-ribosylation factor 3 |
| ATPA_HUMAN | ATP synthase subunit alpha, mitochondrial |
| C1QBP_HUMAN | GC1q-R protein |
| CALD1_HUMAN | Caldesmon |
| CALM_HUMAN | Calmodulin |
| CALR_HUMAN | Calreticulin |
| CALX_HUMAN | Calnexin |
| CAP1_HUMAN | Adenylyl cyclase-associated protein 1 |
| CAZA1_HUMAN | F-actin-capping protein subunit alpha-1 |
| CH60_HUMAN | 60 kDa heat shock protein, mitochondrial |
| CLH1_HUMAN | Clathrin heavy chain 1 |
| CNDP2_HUMAN | Cytosolic non-specific dipeptidase |
| COF1_HUMAN | Cofilin-1 |
| DDX17_HUMAN | Probable ATP-dependent RNA helicase DDX17 |
| DHX9_HUMAN | ATP-dependent RNA helicase A |
| ECHA_HUMAN | Trifunctional enzyme subunit alpha, mitochondrial |
| EF1B_HUMAN | EF-1-beta |
| EF1G_HUMAN | EF-1-gamma |
| EF2_HUMAN | EF-2 |
| ENOA_HUMAN | Alpha-enolase |
| ENPL_HUMAN | Endoplasmin |
| ERO1A_HUMAN | ERO1-like protein alpha |
| EZRI_HUMAN | Ezrin |
| FAS_HUMAN | Fatty acid synthase |
| FERM2_HUMAN | Fermitin family homolog 2 |
| FLNA_HUMAN | Filamin-A |
| G3P_HUMAN | GAPDH |
| GANAB_HUMAN | Neutral alpha-glucosidase AB |
| GDIB_HUMAN | Rab GDP dissociation inhibitor beta |
| GLU2B_HUMAN | Glucosidase 2 subunit beta |
| GRP75_HUMAN | Stress-70 protein, mitochondrial |
| GRP78_HUMAN | 78 kDa glucose-regulated protein |
| GSTP1_HUMAN | Glutathione S-transferase P |
| HNRH1_HUMAN | Heterogeneous nuclear ribonucleoprotein H |
| HNRPC_HUMAN | Heterogeneous nuclear ribonucleoproteins C1/C2 |
| HNRPD_HUMAN | Heterogeneous nuclear ribonucleoproteins C1/C3 |
| HNRPF_HUMAN | Heterogeneous nuclear ribonucleoproteins C1/C4 |
| HNRPK_HUMAN | Heterogeneous nuclear ribonucleoproteins C1/C5 |
| HNRPM_HUMAN | Heterogeneous nuclear ribonucleoproteins C1/C6 |
| HNRPU_HUMAN | Heterogeneous nuclear ribonucleoprotein U |
| HS90A_HUMAN | Heat shock protein HSP 90-alpha |
| HS90B_HUMAN | Heat shock protein HSP 90-beta |
| HSP74_HUMAN | Heat shock 70 kDa protein 4 |
| HSP7C_HUMAN | Heat shock cognate 71 kDa protein |
| HYOU1_HUMAN | Hypoxia up-regulated protein 1 |
| IF2BL_HUMAN | Eukaryotic translation initiation factor 2 subunit 2-like protein |
| IF2B_HUMAN | Eukaryotic translation initiation factor 2 subunit 2 |
| IF4A1_HUMAN | Eukaryotic initiation factor 4A-I |
| ILF3_HUMAN | Interleukin enhancer-binding factor 3 |
| IMA2_HUMAN | Importin subunit alpha-2 |
| K1C10_HUMAN | Keratin, type I cytoskeletal 10 |
| K2C1_HUMAN | Keratin, type II cytoskeletal 1 |
| KPYM_HUMAN | Pyruvate kinase isozymes M1/M2 |
| KU70_HUMAN | Ku70 |
| KU86_HUMAN | ATP-dependent DNA helicase 2 subunit 2 |
| LA_HUMAN | Lupus La protein |
| LDHA_HUMAN | L-lactate dehydrogenase A chain |
| LDHB_HUMAN | L-lactate dehydrogenase B chain |
| LMNA_HUMAN | Prelamin-A/C |
| LPPRC_HUMAN | Leucine-rich PPR motif-containing protein, mitochondrial |
| MATR3_HUMAN | Matrin-3 |
| MYH9_HUMAN | Myosin-9 |
| MYL6_HUMAN | Myosin light polypeptide 6 |
| NACA_HUMAN | Nascent polypeptide-associated complex subunit alpha |
| NASP_HUMAN | Nuclear autoantigenic sperm protein |
| NDKB_HUMAN | Nucleoside diphosphate kinase B |
| NEST_HUMAN | Nestin |
| NONO_HUMAN | Non-POU domain-containing octamer-binding protein |
| NPM_HUMAN | Nucleophosmin |
| NQO1_HUMAN | NAD(P)H dehydrogenase [quinone] 1 |
| NUCL_HUMAN | Nucleolin |
| NUDC_HUMAN | Nuclear migration protein nudC |
| NUDT5_HUMAN | ADP-sugar pyrophosphatase |
| PAIRB_HUMAN | Plasminogen activator inhibitor 1 RNA-binding protein |
| PCBP1_HUMAN | Poly(rC)-binding protein 1 |
| PDIA1_HUMAN | Protein disulfide-isomerase |
| PDIA3_HUMAN | Protein disulfide-isomerase A3 |
| PDIA4_HUMAN | Protein disulfide-isomerase A4 |
| PDIA6_HUMAN | Protein disulfide-isomerase A6 |
| PGAM1_HUMAN | Phosphoglycerate mutase 1 |
| PGK1_HUMAN | Phosphoglycerate kinase 1 |
| PGRC1_HUMAN | Membrane-associated progesterone receptor component 1 |
| PHB2_HUMAN | Prohibitin-2 |
| PPIA_HUMAN | Peptidyl-prolyl cis-trans isomerase A |
| PPIB_HUMAN | Peptidyl-prolyl cis-trans isomerase B |
| PRDX1_HUMAN | Peroxiredoxin-1 |
| PRDX6_HUMAN | Peroxiredoxin-6 |
| PROF1_HUMAN | Profilin-1 |
| PRS4_HUMAN | 26S protease regulatory subunit 4 |
| PSA5_HUMAN | Proteasome subunit alpha type-5 |
| PSD12_HUMAN | 26S proteasome non-ATPase regulatory subunit 12 |
| PTMA_HUMAN | Prothymosin alpha |
| PUR9_HUMAN | Bifunctional purine biosynthesis protein |
| PYRG1_HUMAN | CTP synthase 1 |
| RAB7A_HUMAN | Ras-related protein Rab-7a |
| RL15_HUMAN | 60S ribosomal protein L15 |
| RL4_HUMAN | 60S ribosomal protein L4 |
| RL5_HUMAN | 60S ribosomal protein L5 |
| RL6_HUMAN | 60S ribosomal protein L6 |
| RL7A_HUMAN | 60S ribosomal protein L7a |
| RLA0_HUMAN | 60S acidic ribosomal protein P0 |
| RLA2_HUMAN | 60S acidic ribosomal protein P2 |
| ROA1_HUMAN | Heterogeneous nuclear ribonucleoprotein A1 |
| ROA2_HUMAN | Heterogeneous nuclear ribonucleoproteins A2/B1 |
| RPN1_HUMAN | Dolichyl-diphosphooligosaccharide--protein glycosyltransferase subunit 1 |
| RPN2_HUMAN | Dolichyl-diphosphooligosaccharide--protein glycosyltransferase subunit 2 |
| RS15_HUMAN | 40S ribosomal protein S15 |
| RS18_HUMAN | 40S ribosomal protein S18 |
| RS20_HUMAN | 40S ribosomal protein S20 |
| RS2_HUMAN | 40S ribosomal protein S2 |
| RS3A_HUMAN | 40S ribosomal protein S3a |
| RS3_HUMAN | 40S ribosomal protein S3 |
| RS5_HUMAN | 40S ribosomal protein S5 |
| RSSA_HUMAN | 40S ribosomal protein SA |
| RSU1_HUMAN | Ras suppressor protein 1 |
| SAHH_HUMAN | Adenosylhomocysteinase |
| SERPH_HUMAN | Serpin H1 |
| SET_HUMAN | Protein SET |
| SKP1_HUMAN | S-phase kinase-associated protein 1 |
| STIP1_HUMAN | Stress-induced-phosphoprotein 1 |
| TAGL_HUMAN | Transgelin |
| TBA1B_HUMAN | Tubulin alpha-1B chain |
| TBB5_HUMAN | Tubulin beta chain |
| TCPA_HUMAN | T-complex protein 1 subunit alpha |
| TCPB_HUMAN | T-complex protein 1 subunit beta |
| TCPD_HUMAN | T-complex protein 1 subunit delta |
| TCPE_HUMAN | T-complex protein 1 subunit epsilon |
| TCPG_HUMAN | T-complex protein 1 subunit gamma |
| TCPQ_HUMAN | T-complex protein 1 subunit theta |
| TCPZ_HUMAN | T-complex protein 1 subunit zeta |
| TERA_HUMAN | Transitional endoplasmic reticulum ATPase |
| THOC4_HUMAN | THO complex subunit 4 |
| TIF1B_HUMAN | Transcription intermediary factor 1-beta |
| TKT_HUMAN | Transketolase |
| TMED2_HUMAN | Transmembrane emp24 domain-containing protein 2 |
| TPD54_HUMAN | Tumor protein D54 |
| TPIS_HUMAN | Triosephosphate isomerase |
| TPM4_HUMAN | Tropomyosin alpha-4 chain |
| UAP56_HUMAN | Spliceosome RNA helicase DDX39B |
| UBA1_HUMAN | Ubiquitin-like modifier-activating enzyme 1 |
| UBE2N_HUMAN | Ubiquitin-conjugating enzyme E2 N |
| VDAC1_HUMAN | Voltage-dependent anion-selective channel protein 1 |
| VIME_HUMAN | Vimentin |
| VINC_HUMAN | Vinculin |
| XPO2_HUMAN | Exportin-2 |
| YBOX1_HUMAN | Nuclease-sensitive element-binding protein 1 |
