# Supplementary material for: Identifying New Therapeutic Targets via Modulation of Protein Corona Formation by Engineered Nanoparticles
Source: PLoS One. 2012 Mar 19;7(3):e33650. doi: 10.1371/journal.pone.0033650 (PMC3307759; doi:10.1371/journal.pone.0033650)
Supplement: Table S9 — Comparison of proteins present in −AuNP corona from OSE and OV167 lysates. (DOCX) [file pone.0033650.s012.docx]

**Table S9. Comparison of proteins present in ^-^AuNP corona from OSE and OV167 lysates.**

| **Proteins exclusive to OV 167** | | **Proteins exclusive to OSE** | | | |
| --- | --- | --- | --- | --- | --- |
| Gene Name | Full Name | Gene Name | | Full Name | |
| 6PGD_HUMAN | 6-phosphogluconate dehydrogenase, decarboxylating | ATPO_HUMAN | | ATP synthase subunit O, mitochondrial | |
| ACLY_HUMAN | ATP-citrate synthase | BASP1_HUMAN | | Brain acid soluble protein 1 | |
| ACTB_HUMAN | Actin, cytoplasmic 1 | CAV1_HUMAN | | Caveolin-1 | |
| ANXA6_HUMAN | Annexin A6 | CD44_HUMAN | | CD44 antigen | |
| ARF1_HUMAN | ADP-ribosylation factor 1 | CH10_HUMAN | | 10 kDa heat shock protein, mitochondrial | |
| ARF3_HUMAN | ADP-ribosylation factor 3 | CHRD1_HUMAN | | Cysteine and histidine-rich domain-containing protein 1 | |
| C1QBP_HUMAN | GC1q-R protein | CPNS1_HUMAN | | Calpain small subunit 1 | |
| CALD1_HUMAN | Caldesmon | EF1A1_HUMAN | | Elongation factor 1-alpha 1 | |
| CALM_HUMAN | Calmodulin | EF1A3_HUMAN | | Putative elongation factor 1-alpha-like 3 | |
| CAP1_HUMAN | Adenylyl cyclase-associated protein 1 | FKBP3_HUMAN | | Peptidyl-prolyl cis-trans isomerase FKBP3 | |
| CAZA1_HUMAN | F-actin-capping protein subunit alpha-1 | FKBP4_HUMAN | | Peptidyl-prolyl cis-trans isomerase FKBP4 | |
| CLH1_HUMAN | Clathrin heavy chain 1 | GBG12_HUMAN | | Guanine nucleotide-binding protein | |
| CNDP2_HUMAN | Cytosolic non-specific dipeptidase | H12_HUMAN | | Histone H1.2 | |
| DDX17_HUMAN | Probable ATP-dependent RNA helicase DDX17 | H13_HUMAN | | Histone H1.3 | |
| DHX9_HUMAN | ATP-dependent RNA helicase A | H14_HUMAN | | Histone H1b | |
| ECHA_HUMAN | Trifunctional enzyme subunit alpha, mitochondrial | H2B1B_HUMAN | | Histone H2B type 1-B | |
| EF1B_HUMAN | Elongation factor 1-beta | H2B1C_HUMAN | | Histone H2B type 1-C/E/F/G/I | |
| EF1G_HUMAN | Elongation factor 1-gamma | H2B1D_HUMAN | | Histone H2B type 1-D | |
| EF2_HUMAN | Elongation factor 2 | H2B1H_HUMAN | | Histone H2B type 1-H | |
| ERO1A_HUMAN | ERO1-like protein alpha | H2B1J_HUMAN | | Histone H2B type 1-J | |
| FAS_HUMAN | Fatty acid synthase | H2B1K_HUMAN | | Histone H2B type 1-K | |
| FERM2_HUMAN | Fermitin family homolog 2 | H2B1L_HUMAN | | Histone H2B type 1-L | |
| GANAB_HUMAN | Neutral alpha-glucosidase AB | H2B1M_HUMAN | | Histone H2B type 1-M | |
| GDIB_HUMAN | Rab GDP dissociation inhibitor beta | H2B1N_HUMAN | | Histone H2B type 1-N | |
| GLU2B_HUMAN | Glucosidase 2 subunit beta | H2B1O_HUMAN | | Histone H2B type 1-O | |
| GRP75_HUMAN | Stress-70 protein, mitochondrial | H2B2E_HUMAN | | Histone H2B type 2-E | |
| GSTP1_HUMAN | Glutathione S-transferase P | H2B2F_HUMAN | | Histone H2B type 2-F | |
| HNRH1_HUMAN | Heterogeneous nuclear ribonucleoprotein H | H2B3B_HUMAN | | Histone H2B type 3-B | |
| HNRPC_HUMAN | Heterogeneous nuclear ribonucleoproteins C1/C2 | H2BFS_HUMAN | | Histone H2B type F-S | |
| HNRPD_HUMAN | Heterogeneous nuclear ribonucleoprotein D0 | HMGB1_HUMAN | | High mobility group protein B1 | |
| HNRPF_HUMAN | Nucleolin-like protein mcs94-1 | HSPB1_HUMAN | | Heat shock protein beta-1 | |
| HNRPM_HUMAN | Heterogeneous nuclear ribonucleoprotein M | IQGA1_HUMAN | | Ras GTPase-activating-like protein | |
| HNRPU_HUMAN | Scaffold attachment factor A | ITB1_HUMAN | | Integrin beta-1 | |
| HSP74_HUMAN | Heat shock 70 kDa protein 4 | K1C18_HUMAN | | Cytokeratin-18 | |
| HS90_HUMAN | Heat shock protein 90 | K1C19_HUMAN | | Cytokeratin-19 | |
| HYOU1_HUMAN | Hypoxia up-regulated protein 1 | K1C9_HUMAN | | Cytokeratin-9 | |
| IF2BL_HUMAN | Eukaryotic translation initiation factor 2 subunit 2-like protein | K22E_HUMAN | | Cytokeratin-2e | |
| IF2B_HUMAN | Eukaryotic translation initiation factor 2 subunit 2 | K2C8_HUMAN | | Cytokeratin-8 | |
| IF4A1_HUMAN | Eukaryotic initiation factor 4A-I | LEG1_HUMAN | | 14 kDa lectin | |
| ILF3_HUMAN | Interleukin enhancer-binding factor 3 | MAP4_HUMAN | | Microtubule-associated protein 4 | |
| IMA2_HUMAN | Importin subunit alpha-2 | MARCS_HUMAN | | Myristoylated alanine-rich C-kinase substrate | |
| KU70_HUMAN | Ku70 | MDHC_HUMAN | | Malate dehydrogenase, cytoplasmic | |
| KU86_HUMAN | ATP-dependent DNA helicase 2 | NEDD8_HUMAN | | Neddylin | |
| LA_HUMAN | Lupus La protein | PDCD5_HUMAN | | Programmed cell death protein 5 | |
| LMNA_HUMAN | Prelamin-A/C | PEBP1_HUMAN | | Raf kinase inhibitor protein | |
| LPPRC_HUMAN | Leucine-rich PPR motif-containing protein, mitochondrial | PSME2_HUMAN | | Proteasome activator complex subunit 2 | |
| MATR3_HUMAN | Matrin-3 | RAP1B_HUMAN | | Ras-related protein Rap-1b | |
| NACA_HUMAN | Nascent polypeptide-associated complex subunit alpha | RL12_HUMAN | | 60S ribosomal protein L12 | |
| NASP_HUMAN | Nuclear autoantigenic sperm protein | RL30_HUMAN | | 60S ribosomal protein L30 | |
| NDKB_HUMAN | Nucleoside diphosphate kinase B | RL9_HUMAN | | 60S ribosomal protein L9 | |
| NEST_HUMAN | Nestin | RS10_HUMAN | | 40S ribosomal protein S10 | |
| NONO_HUMAN | Non-POU domain-containing octamer-binding protein | RS17_HUMAN | | 40S ribosomal protein S17 | |
| NPM_HUMAN | Nucleophosmin | RS19_HUMAN | | 40S ribosomal protein S19 | |
| NQO1_HUMAN | NAD(P)H dehydrogenase [quinone] 1 | SUMO2_HUMAN | | Small ubiquitin-related modifier 2 | |
| NUCL_HUMAN | Nucleolin | SYG_HUMAN | | Glycyl-tRNA synthetase | |
| NUDC_HUMAN | Nuclear migration protein nudC | TAGL2_HUMAN | | Transgelin-2 | |
| NUDT5_HUMAN | ADP-sugar pyrophosphatase | TBA1A_HUMAN | | Tubulin alpha-1A chain | |
| PAIRB_HUMAN | Plasminogen activator inhibitor 1 RNA-binding protein | TBB2C_HUMAN | | Tubulin beta-2C chain | |
| PCBP1_HUMAN | Poly(rC)-binding protein 1 | TCTP_HUMAN | | p23 | |
| PDIA3_HUMAN | Protein disulfide-isomerase A3 | TMSL3_HUMAN | | Thymosin beta-4-like protein 3 | |
| PDIA4_HUMAN | Protein disulfide-isomerase A4 | TXND5_HUMAN | | Thioredoxin-like protein p46 | |
| PGAM1_HUMAN | Phosphoglycerate mutase 1 | TYB4_HUMAN | | Thymosin beta-4 | |
| PGRC1_HUMAN | Membrane-associated progesterone receptor component 1 | VAT1_HUMAN | | Synaptic vesicle membrane protein | |
| PHB2_HUMAN | Prohibitin-2 | ZYX_HUMAN | | Zyxin | |
| PPIB_HUMAN | Cyclophilin B |  | |  | |
| PRDX6_HUMAN | Peroxiredoxin-6 |  | |  | |
| PRS4_HUMAN | 26S protease regulatory subunit 4 |  | |  | |
| PSA5_HUMAN | Proteasome subunit alpha type-5 |  | |  | |
| PSD12_HUMAN | 26S proteasome non-ATPase regulatory subunit 12 | | |  | |
| PTMA_HUMAN | Prothymosin alpha | | |  | |
| PUR9_HUMAN | Bifunctional purine biosynthesis protein PURH | | | | |
| PYRG1_HUMAN | CTP synthase 1 |  |  | | |
| RAB7A_HUMAN | Ras-related protein Rab-7a | | | | |
| RL15_HUMAN | 60S ribosomal protein L15 |  |  | | |
| RL4_HUMAN | 60S ribosomal protein L4 |  |  | | |
| RL5_HUMAN | 60S ribosomal protein L5 |  |  | | |
| RL6_HUMAN | 60S ribosomal protein L6 |  |  | | |
| RL7A_HUMAN | 60S ribosomal protein L7a |  |  | | |
| RLA0_HUMAN | 60S acidic ribosomal protein |  |  | | |
| ROA1_HUMAN | Heterogeneous nuclear ribonucleoprotein A1 |  |  | | |
| ROA2_HUMAN | Heterogeneous nuclear ribonucleoproteins A2/B1 | |  | | |
| RPN1_HUMAN | Ribophorin I | | | | |
| RPN2_HUMAN | Ribophorin II | | | | |
| RS15_HUMAN | 40S ribosomal protein S15 |  | | |  |
| RS2_HUMAN | 40S ribosomal protein S2 |  | | |  |
| RS3A_HUMAN | 40S ribosomal protein S3a |  | | |  |
| RS3_HUMAN | 40S ribosomal protein S3 |  | | |  |
| RS5_HUMAN | 40S ribosomal protein S5 |  | | |  |
| RSU1_HUMAN | Ras suppressor protein 1 |  | | |  |
| SAHH_HUMAN | Adenosylhomocysteinase |  | | |  |
| SKP1_HUMAN | S-phase kinase-associated protein 1 |  | | |  |
| STIP1_HUMAN | Stress-induced-phosphoprotein 1 |  | | |  |
| TBA1B_HUMAN | Tubulin alpha-1B chain | | | |  |
| TCPA_HUMAN | T-complex protein 1 subunit alpha | | | |  |
| TCPB_HUMAN | T-complex protein 1 subunit beta |  | | |  |
| TCPD_HUMAN | T-complex protein 1 subunit delta | | | |  |
| TCPE_HUMAN | T-complex protein 1 subunit epsilon | | | |  |
| TCPG_HUMAN | T-complex protein 1 subunit gamma | | | |  |
| TCPZ_HUMAN | T-complex protein 1 subunit zeta | | | |  |
| THOC4_HUMAN | THO complex subunit 4 | | | |  |
| TIF1B_HUMAN | Transcription intermediary factor 1-beta | | | |  |
| TKT_HUMAN | Transketolase |  | | |  |
| TMED2_HUMAN | Membrane protein p24A | | | |  |
| TPM4_HUMAN | Tropomyosin alpha-4 chain |  | | |  |
| UAP56_HUMAN | Spliceosome RNA helicase DDX39B |  | | |  |
| UBA1_HUMAN | Ubiquitin-like modifier-activating enzyme 1 |  | | |  |
| UBE2N_HUMAN | Ubiquitin-conjugating enzyme E2 N | | | |  |
| VDAC1_HUMAN | Voltage-dependent anion-selective channel protein 1 | | | |  |
| VIME_HUMAN | Vimentin | | | |  |
| VINC_HUMAN | Vinculin | | | | |
| XPO2_HUMAN | Exportin-2 |  | | |  |
|  |  |  | | |  |
|  |  |  | | |  |
