# Supplementary material for: Identifying New Therapeutic Targets via Modulation of Protein Corona Formation by Engineered Nanoparticles
Source: PLoS One. 2012 Mar 19;7(3):e33650. doi: 10.1371/journal.pone.0033650 (PMC3307759; doi:10.1371/journal.pone.0033650)
Supplement: Table S10 — Comparison of proteins present in +AuNP corona from OSE and OV167 lysates. (DOCX) [file pone.0033650.s013.docx]

**Table S10. Comparison of proteins present in ^+^AuNP corona from OSE and OV167 lysates.**

| **Proteins exclusive to OV167** | | **Proteins exclusive to OSE** | |
| --- | --- | --- | --- |
| AATM_HUMAN | Aspartate aminotransferase, mitochondrial | 1433T_HUMAN | 14-3-3 protein tau |
| ACTB_HUMAN | Beta-actin | 1433Z_HUMAN | Protein kinase C inhibitor protein 1 |
| ALDOC_HUMAN | Brain-type aldolase | ACTN1_HUMAN | Alpha-actinin-1 |
| CH10_HUMAN | 10 kDa chaperonin | ACTN4_HUMAN | Alpha-actinin-4 |
| CYBP_HUMAN | Calcyclin-binding protein | AT1A1_HUMAN | Sodium/potassium-transporting ATPase subunit alpha-1 |
| ECHA_HUMAN | TP-alpha | ATPA_HUMAN | ATP synthase subunit alpha, mitochondrial |
| FUBP1_HUMAN | DNA helicase V | ATPB_HUMAN | ATP synthase subunit beta, mitochondrial |
| FUBP2_HUMAN | FUSE-binding protein 2 | C1QBP_HUMAN | GC1q-R protein |
| G6PI_HUMAN | Glucose-6-phosphate isomerase | CALR_HUMAN | Calreticulin |
| GDIB_HUMAN | Rab GDI beta | CALU_HUMAN | Calumenin |
| HDGF_HUMAN | Hepatoma-derived growth factor | CALX_HUMAN | Calnexin |
| HNRPQ_HUMAN | Heterogeneous nuclear ribonucleoprotein Q | CAP1_HUMAN | Adenylyl cyclase-associated protein 1 |
| IF5A1_HUMAN | Eukaryotic initiation factor 5A isoform 1 | CAPR1_HUMAN | Caprin-1 |
| LAP2A_HUMAN | Thymopoietin isoform alpha | CKAP4_HUMAN | Cytoskeleton-associated protein 4 |
| LAP2B_HUMAN | Thymopoietin, isoforms beta/gamma | CLH1_HUMAN | Clathrin heavy chain 1 |
| MDHM_HUMAN | Malate dehydrogenase, mitochondrial | CO1A1_HUMAN | Collagen alpha-1(I) chain |
| NACA_HUMAN | NAC-alpha | DYHC1_HUMAN | Dynein heavy chain |
| NEST_HUMAN | Nestin | EF1A1_HUMAN | Elongation factor Tu |
| NQO1_HUMAN | NAD(P)H dehydrogenase | EF1A3_HUMAN | EF-1-alpha-like 3 |
| NUCKS_HUMAN | Nuclear ubiquitous casein | EF1G_HUMAN | EF-1-gamma |
| PA2G4_HUMAN | Proliferation-associated protein 2G4 | ENPL_HUMAN | Endoplasmin |
| PAIRB_HUMAN | PAI1 RNA-binding protein 1 | FAS_HUMAN | Fatty acid synthase |
| PARK7_HUMAN | Protein DJ-1 | FLNB_HUMAN | Filamin-B |
| PARP1_HUMAN | Poly [ADP-ribose] polymerase 1 | FLNC_HUMAN | Filamin-C |
| PDIA6_HUMAN | Protein disulfide-isomerase A6 | GANAB_HUMAN | Neutral alpha-glucosidase AB |
| PGAM1_HUMAN | Phosphoglycerate mutase 1 | GLSK_HUMAN | Glutaminase kidney isoform, mitochondrial |
| PROF1_HUMAN | Profilin-1 | GLU2B_HUMAN | Glucosidase 2 subunit beta |
| PTBP1_HUMAN | Polypyrimidine tract-binding protein 1 | HORN_HUMAN | Hornerin |
| RS3A_HUMAN | 40S ribosomal protein S3a | HS105_HUMAN | Heat shock protein 105 kDa |
| RSU1_HUMAN | Ras suppressor protein 1 | HSPB1_HUMAN | Heat shock protein beta-1 |
| S10A4_HUMAN | Protein S100-A4 | IF4A1_HUMAN | eIF-4A-I |
| STIP1_HUMAN | Stress-induced-phosphoprotein 1 | IF4G1_HUMAN | eIF-4-gamma 1 |
| TBA1B_HUMAN | Tubulin alpha-1B chain | IMA2_HUMAN | Importin subunit alpha-2 |
| VINC_HUMAN | Vinculin | IMB1_HUMAN | Importin subunit beta-1 |
| YBOX1_HUMAN | DNA-binding protein B | IPO5_HUMAN | Importin-5 |
|  |  | IQGA1_HUMAN | p195 |
|  |  | LEG1_HUMAN | Galaptin |
|  |  | LMNA_HUMAN | Prelamin-A/C |
|  |  | MARCS_HUMAN | MARCKS |
|  |  | MYH9_HUMAN | Myosin-9 |
|  |  | MYL6_HUMAN | Myosin light polypeptide 6 |
|  |  | NP1L4_HUMAN | Nucleosome assembly protein 2 |
|  |  | PDIA4_HUMAN | Protein disulfide-isomerase A4 |
|  |  | PLEC1_HUMAN | Plectin |
|  |  | PLST_HUMAN | Plastin-3 |
|  |  | PPIA_HUMAN | Cyclophilin A |
|  |  | PRKDC_HUMAN | DNA-dependent protein kinase catalytic subunit |
|  |  | PTRF_HUMAN | Polymerase I and transcript release factor |
|  |  | RHOA_HUMAN | Transforming protein RhoA |
|  |  | RINI_HUMAN | Ribonuclease inhibitor |
|  |  | SAHH_HUMAN | Adenosylhomocysteinase |
|  |  | SET_HUMAN | Protein SET |
|  |  | TAGL2_HUMAN | Transgelin-2 |
|  |  | TBA1A_HUMAN | Alpha-tubulin 3 |
|  |  | TBB2C_HUMAN | Tubulin beta-2C chain |
|  |  | TBB6_HUMAN | Tubulin beta-6 chain |
|  |  | TCPA_HUMAN | CCT-alpha |
|  |  | TCPD_HUMAN | TCP-1-delta |
|  |  | TCPE_HUMAN | TCP-1-epsilon |
|  |  | TCPG_HUMAN | TCP-1-gamma |
|  |  | TCPH_HUMAN | TCP-1-eta |
|  |  | TCPQ_HUMAN | TCP-1-theta |
|  |  | TCPZ_HUMAN | TCP-1-zeta |
|  |  | TCTP_HUMAN | Translationally-controlled tumor protein |
|  |  | TERA_HUMAN | Valosin-containing protein |
|  |  | TIF1B_HUMAN | TIF1-beta |
|  |  | TLN1_HUMAN | Talin-1 |
|  |  | TPM2_HUMAN | Beta-tropomyosin |
|  |  | TPM4_HUMAN | Tropomyosin-4 |
|  |  | TRXR1_HUMAN | Thioredoxin reductase |
|  |  | UBA1_HUMAN | Ubiquitin-activating enzyme E1 |
|  |  | XPO1_HUMAN | Exportin-1 |
|  |  | XPO2_HUMAN | Exportin-2 |
